# Plasma Protein Pattern Correlates With Pain Intensity and Psychological Distress in Women With Chronic Widespread Pain

**DOI:** 10.3389/fpsyg.2018.02400

**Published:** 2018-11-29

**Authors:** Karin Wåhlén, Bijar Ghafouri, Nazdar Ghafouri, Björn Gerdle

**Affiliations:** Pain and Rehabilitation Centre, Department of Medical and Health Sciences, Linköping University, Linköping, Sweden

**Keywords:** biomarker, fibromyalgia, pain, psychological distress, inflammation

## Abstract

**Objectives:** Although generalized muscle pain, tiredness, anxiety, and depression are commonly present among chronic widespread pain (CWP) patients, the molecular mechanisms behind CWP are not fully elucidated. Moreover, the lack of biomarkers often makes diagnosis and treatment problematic. In this study, we investigated the correlation between pain intensity, psychological distress, and plasma proteins among CWP patients and controls (CON).

**Methods:** The plasma proteome of CWP (*n* = 15) and CON (*n* = 23) was analyzed using two-dimensional gel electrophoresis. Orthogonal Partial Least Square analysis (OPLS) was used to determine proteins associated with pain intensity (numeric rating scale) in CWP and psychological distress (Hospital and Depression Scale, HADS) in CWP and CON. Significant proteins were identified by MALDI-TOF and tandem MS.

**Results:** In CWP, pain intensity was associated with plasma proteins mostly involved in metabolic and immunity processes (e.g., kininogen-1, fibrinogen gamma chain, and ceruloplasmin), and psychological distress was associated with plasma proteins related to immunity response, iron ion, and lipid metabolism (e.g., complement factor B, complement C1r subcomponent, hemopexin, and clusterin).

**Discussion:** This study suggests that different plasma protein patterns are associated with different pain intensity and psychological distress in CWP. Proteins belonging to the coagulation cascade and immunity processes showed strong associations to each clinical outcome. Using the plasma proteome profile of CWP to study potential biomarker candidates provides a snapshot of ongoing systemic mechanisms in CWP.

## Introduction

Chronic widespread pain (CWP), including fibromyalgia syndrome (FMS), is a complex pain condition characterized by generalized musculoskeletal pain and is often associated with symptoms such as tiredness, sleep disturbance, depression, anxiety, and cognitive difficulties (Wolfe et al., 1990; Aparicio et al., 2013; Perez de Heredia-Torres et al., 2016). Chronic pain not only affects the patients but also affects their families and society, leading to extensive suffering and high economic costs (Breivik et al., 2006).

Diagnosis of CWP is based on clinical examinations and criteria included in the American College of Rheumatology (ACR 90) definition of FMS (Wolfe et al., 1990). Hence, CWP means that pain has to be chronic (>3 months duration) and widespread (i.e., in the spine and in at least three out of four defined body quadrants or in the spine and in contralateral quadrants). Diagnosis of FMS also requires generalized hyperalgesia (examined using tender point examination). In the European adult population, the prevalence of CWP is about 10%, with a higher prevalence for females (Bergman et al., 2001; Breivik et al., 2006; Cimmino et al., 2011; Mansfield et al., 2016). Furthermore, the variety of symptoms among CWP and the complex multifactorial etiology make it difficult to study the biological mechanisms behind CWP, mechanisms that need further elucidation. Although both central and peripheral mechanisms may contribute to the perceived pain in CWP, (Staud et al., 2001, 2009; Flodin et al., 2014) no valid biological markers have been identified for the activated nociceptive mechanisms in chronic pain conditions, including CWP. This and other studies from our group focus on exploring such activated mechanisms with the long-term goal of identifying clinically applicable biomarkers that can facilitate mechanism-based diagnoses and choice of treatments. Many biomarker studies have analyzed protein patterns, cytokines/chemokines, lipids, and metabolites in plasma/serum, muscles, and saliva to understand activated nociceptive mechanisms in patients with CWP/FMS (Bazzichi et al., 2009; Zanette et al., 2014; Hadrevi et al., 2015; Culic et al., 2016; Gerdle et al., 2017; Olausson et al., 2017; Wåhlén et al., 2017). Taken together, these studies clearly indicate that peripheral (muscle and/or blood) nociceptive and inflammatory mechanisms are active and differ between patients and healthy controls.

One important aspect of pain both clinically and in research is pain intensity, which is registered using self-reported pain scales such as numeric rating scale (NRS) or visual analog scale (VAS) (Farrar et al., 2001; Ferreira-Valente et al., 2011; Kliger et al., 2015). Recently, we reported that pain intensity correlated significantly with the muscle protein pattern in CWP (Olausson et al., 2016) and that several of the identified significant proteins were involved in stress and inflammatory responses and metabolic pathways. However, it is not known whether such a relationship exists between pain intensity and the protein pattern in blood. As mentioned above, psychological distress is common in CWP and is often measured using the Hospital Anxiety and Depression Scale (HADS) (Zigmond and Snaith, 1983). Because the association between depression and several serum proteins has been shown in patients with major depression disorder, (Lee et al., 2015; Ruland et al., 2016) it is important to investigate whether the protein pattern in blood correlates with psychological distress in CWP patients and to what extent these proteins influence pain intensity. The same principal reasoning goes for Body Mass Index (BMI) since increased BMI is often found in patients with CWP/FMS (Neumann et al., 2008) and the correlation between limited numbers of plasma/serum metabolites and proteins and BMI have been reported (Xiao et al., 2013; Rus et al., 2016).

Using proteomics to investigate disease specific markers in plasma and serum is common in the cardiovascular, cancer, and neurodegenerative research (Geyer et al., 2017). There are at least two advantages with analyzing plasma: its rich protein content is easily accessed and since blood is in direct contact with all tissues, changes in peripheral tissues can be easily detected (Anderson and Anderson, 2002). However, plasma proteomic studies comprise several challenges. Plasma contains a complex mixture of proteins with large dynamic range, dominated by high abundant proteins such as albumin and immunoglobulins (Anderson and Anderson, 2002). Multidimensional fractionation or depletion techniques are required to sufficiently reduce sample complexity (Cao et al., 2012). Mass spectrometry in combination with various separation methods such as two-dimensional gel electrophoresis (2-DE) and liquid chromatography (LC) can identify thousands of proteins in a single analysis, and this information can be used to study how proteins are expressed and regulated (Gorg et al., 2009; Westermeier and Gorg, 2011). This strategy has successfully been applied in multiple quantitative proteomic studies of the cerebral spinal fluid (CSF) and serum of subjects with chronic low back pain (with and without pain related to lumbar disk hernia) (Liu et al., 2006; Zhang et al., 2014). Compared to other separation methods, 2-DE provides a high quality of protein resolution as shown by its capability to resolve many post-translationally modified proteins that appear as isoforms.

Previously, we used 2-DE to investigate the plasma proteome between CWP patients and healthy controls (Wåhlén et al., 2017). We found several proteins belonging to inflammatory, immunity, and metabolic processes that could discriminate the CWP and CON group. Based on these results, we now want to investigate the correlation between the altered proteins and pain intensity, depression, and anxiety. To the best of our knowledge, this type of investigation has not been reported. The analysis of the plasma proteome and investigation of associated symptoms in CWP patients using proteomics could improve the knowledge of the activated biological mechanisms in CWP.

Here, we investigated the correlation between pain intensity, psychological distress, and plasma proteins among CWP patients and controls and analyzed the possible influences of BMI and age on such relationships.

## Materials and Methods

### Subjects

The recruitment process, including inclusion and exclusion criteria, for CWP patients and healthy controls (CON) has previously been described in detail (Gerdle et al., 2014). None of the included subjects used any type of opioid, steroidal, or anticoagulatory medication. Exclusion criteria also included medical history record of bursitis, tendonitis, capsulitis, post-operative conditions in the neck/shoulder area, previous neck trauma, disorder of the spine, neurological disease, rheumatoid arthritis or any other systemic diseases, metabolic disease, malignancy, severe psychiatric illness or pregnancy, or difficulties understanding the Swedish language.

The healthy CON group consisted of women between 20 and 65 years. They were recruited through local newspaper advertisements. Women with CWP were recruited from former patients with CWP at the Pain and Rehabilitation Centre of the University Hospital, Linköping, Sweden and from an organization for FMS patients. As reported in previous studies, (Gerdle et al., 2010, 2014; Ghafouri et al., 2013) a total of 19 CWP and 24 CON were initially recruited in the original study. However, four participants were not used because there were difficulties collecting blood samples (two CWP subjects) and because two plasma samples (one CWP and one CON) were insufficient for further proteomic analysis. This resulted in 16 CWP and 23 CON samples. Hence, the proteomic data that this present study is based on has previously been published (Wåhlén et al., 2017). However, in this present study, one of the CWP patients was excluded due to unclear diagnosis after detailed analysis and due to the fact that this patient had incomplete data in the health questionnaire. This exclusion resulted in statistical analysis of 38 plasma samples in this present study, 15 plasma samples from CWP and 23 from CON.

To confirm the individual eligibility, all participants (CWP and CON) received a standardized clinical examination. The ACR 90 criteria were used for classification of FMS/CWP (Wolfe et al., 1990). The recruiting process started in January 2010 and finished in May 2011. Hence, the revised ACR criteria from 2016 was not available. The examination was followed by a health questionnaire (see below). At the clinical examination, weight and height were registered. Based on these measurements, BMI (kg/m^2^) was calculated as weight (kg)/height (m)^2^ and classified according to the criteria developed by the World Health Organization (WHO): < 18.5 = underweight; 18.5–24.9 = normal range; 25.0–29.9 = overweight; and ≥30.0 = obesity.

All participants signed a written consent form before the start of the study after receiving verbal and written information about the objectives and procedures of the study. The study was approved by the Regional Ethical Review Board in Linköping, Sweden (Dnr: M10–08, M233–09, Dnr: 2010/164–32) and followed the guidelines according to the Declaration of Helsinki. All methods were carried out in accordance with the approved ethical application.

## Methods

All subjects answered a health questionnaire consisting of the following items and scales.

### Demographic Data

Each subject reported age (years).

### Pain Intensity and Duration

Each subject rated the pain intensity in the neck-shoulder region, low back and whole body using an 11 grade (0 – 10) NRS with two endpoints: zero indicating no pain at all and 10 indicating worst possible pain (Ferreira-Valente et al., 2011). CWP patients also reported the pain duration (years).

### Hospital Anxiety and Depression Scale (HADS)

The HADS is a short self-assessment questionnaire that measures anxiety and depression (Zigmond and Snaith, 1983). HADS comprises seven items in each of the depression and anxiety scales (HAD-Depression and HAD-Anxiety). The subscale scores range between 0 and 21, with the lower score indicating the least depression and anxiety possible (Zigmond and Snaith, 1983). HADS is frequently used both in clinical practice and in research and has good psychometric characteristics (Zigmond and Snaith, 1983; Bjelland et al., 2002). It is also validated in its Swedish translation (Lisspers et al., 1997). In this study, a total score of HADS (denoted HADS-total), which includes both the anxiety and depression scores, was used to indicate psychological distress.

### Other Background Variables

To get a comprehensive description of the subjects also data from the Pain Catastrophizing Scale (PCS) and Quality of Life instrument (QoL) are reported; for details about these instruments see our previous studies (Gerdle et al., 2014; Wåhlén et al., 2017).

### Sample Collection

Before blood sampling, all participants were asked not to take any non-steroidal anti-inflammatory drugs for 7 days and/or paracetamol medication 12 h before the sampling. Venous blood samples were collected in EDTA vacutainer and centrifuged at 1000 × *g* for 15 min. The plasma was collected, aliquoted, and stored at −70°C. All samples were blinded before analysis.

### Two-Dimensional Gel Electrophoresis (2-DE)

The procedure for 2-DE, including sample preparation, has previously been described in detail (Gorg et al., 2009; Olausson et al., 2015; Wåhlén et al., 2017). In brief, depleted plasma samples containing 100 μg total protein were run in the first dimension, followed by second dimension separation using Ettan^TM^ DALTsix Electrophoresis Unit (Amersham, Pharmacia, Uppsala, Sweden). The protein gels were fluorescently stained using SYPRO Ruby^®^ (Bio-Rad Laboratories, Hercules, CA, United States). The stained protein pattern was visualized using a charge coupled device camera (VersaDoc^TM^ Imaging system 4000 MP, Bio-Rad) and further analyzed and quantified using PDQuest Advanced (v. 8.0.1, Bio-Rad). The amount of protein in a spot was assessed as background-corrected optical density, integrated over all pixels in the spot, and expressed as integrated optical density (IOD). Quantified protein data were then analyzed with multivariate statistics. The coefficient of variation of 2-DE was less than 25%, which is in line with what others have found with 2-DE (Magdeldin et al., 2014). Two preparative gels (one pool from CWP and one from CON, containing 400 μg of total protein) for protein identifications were run according to the above protocol.

### Protein Identification

For identification, protein spots of interest were excised from the preparative gels, de-stained, subjected to tryptic digestion, and prepared as previously described (Olausson et al., 2015). Briefly, the gel piece was incubated in 50% acetonitrile (ACN) in 25 mM ammonium bicarbonate, dehydrated in 100% ACN, dried in SpeedVac, and trypsinated in 37°C over night. The supernatant was transferred to a new tube, and the peptides were further extracted from the gel piece by incubation of 5% trifluoroacetic acid (TFA) in 50% ACN for 4 h. The pooled supernatants were dried and stored at −20°C until analysis.

Briefly, for MALDI-TOF analysis, peptides were reconstituted in 4 μl 0.1% TFA. The peptides were mixed in a 1:1 ratio with matrix solution (dihydroxybenzoic acid in 70% acetonitrile/0.3% TFA) and 1 μl was spotted on a target plate (stainless steel). The peptide masses were analyzed and the mass range of 300–3500 Da was used, including external mass calibration using a peptide calibration standard (Bruker) (Olausson et al., 2017).

Low abundant proteins were identified with a nano liquid chromatography system (EASY-nLC, Thermo Scientific, Waltham, MA, United States) coupled to an LTQ Orbitrap Velos Pro MS (Thermo Scientific). The same procedure for LC-MS analysis was used as described earlier with minor adjustments in time (Olausson et al., 2017). Peptides were dissolved in 6 μl 0.1% formic acid (FA) and loaded on a C18 column (100 mm × 75 μM, particle size 5 μM). The flow rate was set to 300 nL/min and the gradient buffer contained 0.1% FA in water (buffer A) and 0.1% FA in ACN (buffer B). Buffer B was used in a linear gradient (0–100%) for 30 min to separate the peptides.

### Database Search and Bioinformatics

The acquired MS data from MALDI-TOF analysis was pre-processed using flexAnalysis v. 3.4 (Bruker Daltonik), and the major peak list from each processed spectra was imported in the search engine ProteinProspector MS-Fit (v. 5.14.4), including the Swiss-Prot database v. 2015.3.5, as described in previous studies (Olausson et al., 2017; Wåhlén et al., 2017). Parameter restriction was made based on species (*Homo sapiens*), mass tolerance (50 ppm), maximum miss cleavages by trypsin (≤1), fixed modifications (carbamidomethylation of cysteine), and possible dynamic modifications (oxidation of methionine).

The acquired MS data from the Orbitrap were analyzed with MaxQuant v. 1.5.8.3 (Max Planck Institute of Biochemistry, Martinsried, Germany) using the human UniProt/Swiss-Prot database (downloaded 20170404) as described previously (Olausson et al., 2017). The analysis parameters were as follow: mass tolerance (0.5 Da), parent ion tolerance (6 ppm), miss cleavages by trypsin (maximum 2), fixed modifications (carbamidomethylation of cysteine), and variable modification (oxidation on methionine and N-terminal acetylation). A false discovery rate of <1% was used and at least two unique peptides were needed to be considered as identified. The identified proteins were divided in different groups based on UniProt database ^1^ definition on biological process.

### Statistics

#### Univariate Statistics

For comparison of group differences regarding clinical background data, pain intensity, and HADS, Student’s *t*-test and the non-parametric Mann–Whitney *U*-test were applied (IBM SPSS v. 24.0, IBM, United States) for normal distributed data and for non-normally distributed data, respectively, *p* < 0.05 was considered significant.

#### Multivariate Data Analysis (MVDA)

To investigate the multivariate correlations between the proteins (*X*-variables) and the clinical variables (*Y*-variables), OPLS was applied using SIMCA-P+ v. 13.0 (UMETRICS, Umeå, Sweden) (Eriksson et al., 2006). When applying MVDA, we followed the recommendations concerning omics data presented by Wheelock and Wheelock (Wheelock and Wheelock, 2013). The procedure of MVDA has been described in detail elsewhere (Olausson et al., 2015, 2017; Wåhlén et al., 2017).

Briefly, PCA was used before all OPLS analysis in order to check for multivariate outliers. Furthermore, OPLS was used for the regression analyses of pain intensity, depressive and anxiety symptoms (HADS-total), BMI, and age using the detected proteins as regressors (*X*-variables) and clinical variables as *Y*-variables. All variables were mean centered, scaled for unified variance (UV-scaling), and transformed (log) if necessary (Eriksson et al., 2006). Variable influence on projection (VIP) value >1.0 combined with jack-knifed 95% confidence intervals in the regression coefficients plot not including zero were considered significant. In the present study, the analysis was made in two steps. First, all proteins were included and from this analysis selected proteins with VIP > 1.0 were used in a new regression presented in the results. Second, the significant (VIP > 1.0) proteins were identified. In the tables, p(corr) is presented for each significant variable. This is the loading of each variable scaled as a correlation coefficient and thus standardizing the range from −1 to +1 (Wheelock and Wheelock, 2013). Furthermore, for each OPLS model, R^2^ and Q^2^ are displayed describing the goodness of fit and goodness of prediction of each model (Eriksson et al., 2006). To validate the model, we used cross validated analysis of variance (CV-ANOVA), and *p* ≤ 0.05 was considered a significant model. All presented variables are in accordance with Wheelock and Wheelock (Wheelock and Wheelock, 2013).

## Results

### Clinical Background Data

No significant differences were found between CWP and CON regarding height, weight, and BMI. The CWP group was significantly older and reported significantly higher HADS-total compared to CON. As expected, the pain intensity, as measured by NRS, was significantly higher in the CWP group. CWP also reported lower quality of life as well as more catastrophizing thoughts (Table 1).

**Table 1:** Demographic data, clinical measurements of pain intensity (NRS) and other pain characteristics, presented as mean values (±1 standard deviation) and median (min–max).

Variables	CON (*n* = 23)		CWP (*n* = 15)		*p*-value
	Mean ± SD	Median (min–max)	Mean ± SD	Median (min–max)	
Age (years)	41.0 ± 10.2	42.0(27–56)	49.2 ± 8.9	50.0(31–62)	0.015^a^
Height (cm)	168.7 ± 7.3	169(153–181)	167.1 ± 5.0	169(156–173)	0.473^b^
Weight (kg)	68.7 ± 11.2	69.0(50–100)	72.8 ± 15.9	68.0(53–105)	0.351^b^
BMI (kg/m^2^)	24.0 ± 2.8	23.2(19.5–31.9)	26.0 ± 5.0	23.8 (20–35.6)	0.133^b^
Pain duration (years)	NA	NA	12.9 ± 8.4	10.0(4.0–34)	NA
NRS (whole body)	0.0 ± 0.0	0.0(0.0–0.0)	4.9 ± 2.0	5.0(1.0–8.0)	< 0.001^b^
Pain the last 7 days					
Neck (NRS)	0.1 ± 0.4	0.0(0.0–2.0)	5.7 ± 2.4	6.0(0.0–9.0)	< 0.001^b^
Shoulders (NRS)	0.0 ± 0.0	0.0(0.0–0.0)	5.7 ± 1.9	6.0(2.0–8.0)	< 0.001^b^
Low back (NRS)	0.0 ± 0.0	0.0(0.0–0.0)	5.9 ± 1.6	6.0(3.0–9.0)	< 0.001^b^
HADS depression	1.3 ± 1.6	1.0(0.0–6.0)	6.1 ± 3.4	6.0(1.0–13)	< 0.001^a^
HADS anxiety	1.9 ± 1.9	1.0(0.0–7.0)	7.9 ± 3.2	6.0(5.0–14)	< 0.001^a^
HADS total	3.3 –2.8	2.0(0.0–9.0)	14.0 ± 5.3	13.0(7.0–24)	< 0.001^a^
PCS	6.7 ± 6.4	5.0(0.0–19.0)	13.0 ± 7.5	13.0(4.0–29)	0.011^a^
QoL	93.1 ± 9.7	94(74–111)	82.5 ± 13.1	85.0(57–103)	0.007^b^
Also diagnosed with FM (*n*)	NA	NA	13	NA	NA

*Statistical test used: ^a^Mann–Whitney U-test, ^b^Student t-test. NRS, numeric rating scale; BMI, body mass index; HADS, hospital Anxiety and depression scale; SD, standard deviation; CON, control; CWP, chronic widespread pain; NA, Not applicable; PCS, pain catastrophizing scale; QoL, Quality of life; FM, fibromyalgia. Please note that these data (not the exact same number of patients) have been published earlier (Wåhlén et al., 2017).*

### 2-DE Analysis

A total of 414 ± 21 (CWP: 425 ± 18, CON: 408 ± 20) plasma proteins, including different isoforms (in the following termed proteoforms) from each gel, were detected in the 2-DE analysis, and 325 proteins were further quantified, matched, and analyzed with OPLS models. The quantified proteins were initially analyzed with an unsupervised PCA to detect outliers. In this study, no moderate or strong outliers were found. Most of the significantly protein spots were identified in previous study (Wåhlén et al., 2017). There were 57 proteins that were not identified previously and these spots were excised from the gel and identified by mass spectrometry (Supplementary Table S1 and Supplementary Figure S1).

### OPLS Models

In total, seven OPLS models were created to analyze the correlation between expressed plasma proteins and NRS, HADS-total, BMI, and age in CWP and CON. The following protein distributions in all models were found – metabolic: CWP = 41% and CON = 35%; immunity: CWP = 30% and CON = 37%; iron ion homeostasis: CWP = 8% and CON = 8%; inflammatory: CWP = 4% and CON = 15%; lipid metabolism: CWP = 18% and CON = 3%; and unknown processes: CWP = 0% and CON = 2%.

### Plasma Proteins in Relation to Pain Intensity (NRS) in the CWP Group

**FIGURE 1:**
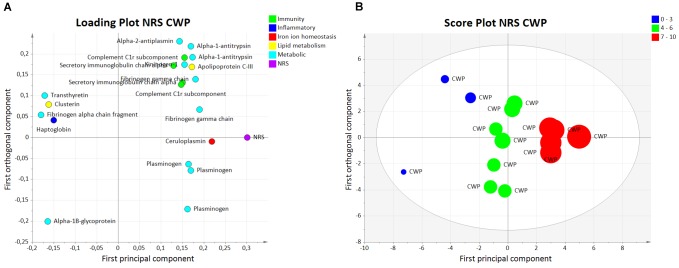
Orthogonal partial least squares regression analysis model of NRS in CWP. **(A)** Loading plot showing proteins with VIP value >1.0 significantly associated to pain intensity, e.g., fibrinogen gamma chain, ceruloplasmin, and plasminogen. The proteins are grouped based on biological process. **(B)** Score plot illustrating separation within the CWP group based on reported pain intensity: mild (0–3), moderate (4–6), and severe (7–10). NRS, numeric rating scale; CWP, chronic widespread pain; OPLS, orthogonal partial least squares regression analysis.

The OPLS model of NRS (NRS_CWP_) consisted of one predictive and one orthogonal component with a high sensitivity (*R*^2^ = 0.97), predictivity (*Q*^2^ = 0.85), and a significant CV-ANOVA (*p*-value < 0.001). A total of 20 proteins had a VIP value >1.0 and were considered significant regressors for NRS (*Y*-variable) in the CWP (Figure 1A). In the score plot of NRS_CWP_ (Figure 1B), a separation within the group is seen based on the respective NRS value, representing mild (0–3), moderate (4–6), and severe (7–10) pain intensity. The majority of the significant proteins belonged to metabolic and immunity processes, and other proteins belonged to lipid metabolism, iron ion homeostasis, and inflammatory processes (Table 2).

**Table 2:** Orthogonal partial least squares regression analysis model of pain intensity (NRS) in CWP group.

Spot number	Protein name	Accession number	Biological process	Experimental MW (kDa)/p*I*	VIP	p(corr)	OD CON (Mean ± SD)	OD CWP (Mean ± SD)	OD quotient mean	Alteration CWP vs. CON
**3817**	**Ceruloplasmin**	**P00450**	**Iron ion homeostasis**	**163 / 5.41**	**1.40**	**0.74**	**62** ± **103**	**184** ± **161**	**2.97**	**↑**
**4304**	**Fibrinogen gamma chain**	**P02679**	**Metabolic**	**71 / 5.66**	**1.32**	**0.64**	**16721** ± **6137**	**25746** ± **8738**	**1.54**	**↑**
**2701**	**Alpha-1B-glycoprotein**	**P04217**	**Metabolic**	**109 / 5.32**	**1.26**	−**0.55**	**1539** ± **558**	**1622** ± **361**	**1.05**	**↑**
**4302**	**Fibrinogen gamma chain**	**P02679**	**Metabolic**	**71 / 5.58**	**1.19**	**0.61**	**11371** ± **3908**	**17521** ± **5893**	**1.54**	**↑**
**510**	**Kininogen-1**	**P01042**	**Metabolic**	**87 / 4.88**	**1.18**	**0.52**	**440** ± **560**	**854** ± **725**	**1.94**	**↑**
8822	Plasminogen	P00747	Metabolic	143 / 6.91	1.15	0.55	430 ± 292	745 ± 337	1.73	**↑**
1052	Apolipoprotein C-III	P02656	Lipid metabolism	8 / 4.22	1.15	0.58	774 ± 910	1013 ± 1355	1.31	**↑**
9901	Plasminogen	P00747	Metabolic	143 / 7.11	1.14	0.54	570 ± 332	763 ± 313	1.34	**↑**
4809^∗^	Complement C1r subcomponent	P00736	Immunity	132 / 5.58	1.13	0.52	158 ± 101	238 ± 155	1.51	**↑**
8909	Plasminogen	P00747	Metabolic	151 / 6.65	1.12	0.57	322 ± 203	618 ± 375	1.92	**↑**
2116^∗^	Transthyretin	P02766	Iron ion homeostasis	13 / 4.75	1.10	−0.57	3497 ± 2539	1265 ± 1382	0.36	**↓**
4316^∗^	Haptoglobin	P00738	Inflammatory	60 / 5.47	1.10	−0.51	12243 ± 5754	12341 ± 7702	1.01	**↑**
4807	Secretory immunoglobulin chain alpha	P99003	Immunity	125 / 5.51	1.09	0.51	114 ± 101	149 ± 117	1.31	**↑**
9208	Fibrinogen alpha chain fragment	P02671	Metabolic	40 / 8.07	1.09	−0.61	509 ± 315	504 ± 390	0.99	**↓**
4810	Complement C1r subcomponent	P00736	Immunity	132 / 5.63	1.07	0.50	247 ± 174	569 ± 239	2.30	**↑**
2903^∗^	Alpha-1 -antitrypsin	P01009	Metabolic	151 / 5.32	1.06	0.58	57 ± 111	70 ± 47	1.23	**↑**
2604	Alpha-2 -antiplasmin	P08697	Metabolic	103 / 5.36	1.05	0.48	166 ± 185	265 ± 212	1.60	**↑**
4713	Secretory immunoglobulin chain alpha	P99003	Immunity	125 / 5.47	1.05	0.43	135 ± 124	129 ± 119	0.96	**↓**
114	Clusterin	P10909	Lipid metabolism	46 / 4.99	1.05	−0.54	1879 ± 1725	1621 ± 921	0.86	**↓**
2904	Alpha-1 -antitrypsin	P01009	Metabolic	151 / 5.36	1.04	0.57	64 ± 97	99 ± 67	1.55	**↑**

*20 proteins had a VIP > 1. The proteins with highest VIP value (bold) were considered as important regressors for the model and belongs to metabolic and Iron ion homeostasis processes.^∗^Shared proteoforms with OPLS model of BMI_CON_ and Age_CON_. NRS, numeric rating scale; SD, standard deviation; CON, control; CWP, chronic widespread pain; OD, optical density; MW, molecular weight; pI, isoelectric point; VIP, variable influence on projection; OPLS, orthogonal partial least squares regression analysis.*

**FIGURE 2:**
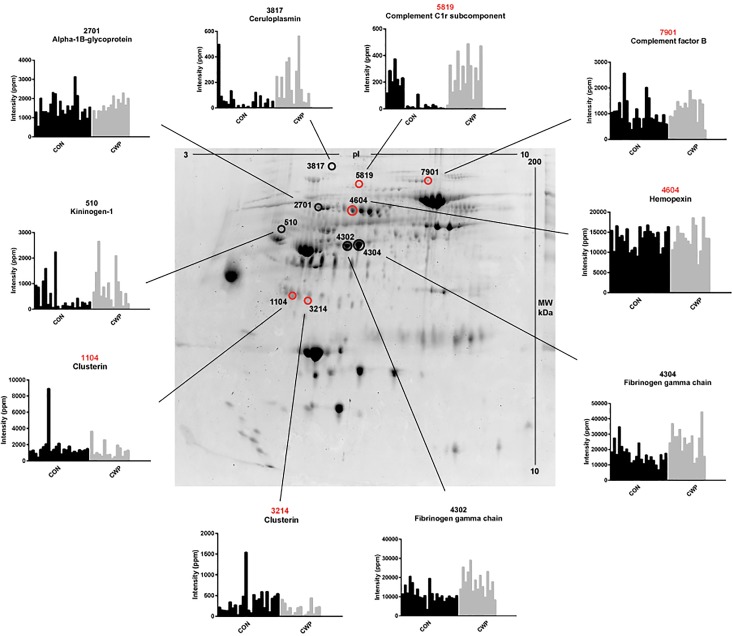
Plasma protein pattern and significant proteoforms from the OPLS models of NRS and HADS-total CWP. The preparative 2-DE gel (used for protein identification) was used to visualize the plasma proteome. Marked proteoforms represent the five most important proteins with highest VIP value for the OPLS model in NRS (marked in black) and HADS-total in CWP (marked in red). The quantified intensity (*Y*-axis) of the proteoform in individual samples (*X*-axis) in CWP and CON group is illustrated in the diagrams. The numbers refer to equal numbers in each table of the respective model. NRS, numeric rating scale; CWP, chronic widespread pain; CON, control; HADS, hospital anxiety and depression scale; VIP, variable influence on projection; OPLS, orthogonal partial least squares regression analysis; PPM, part per million.

The five proteins with the highest VIP values (VIP > 1.18) and strongest associations with NRS were ceruloplasmin (iron ion homeostasis process), alpha-1B-glycoprotein, kininogen-1, and two proteoforms of fibrinogen gamma chain (metabolic processes) (Table 2). These proteoforms are shown on a 2-DE gel (marked in black) to visualize the plasma protein pattern and the quantified intensity from individual samples in each group (Figure 2). The spot numbers on the gel correspond to the spot numbers in Table 2.

### Plasma Proteins Versus HADS-Total

#### CWP

The OPLS model for HADS (HADS_CWP_) consisted of one predictive and one orthogonal component with a high sensitivity (*R*^2^ = 0.96), predictivity (*Q*^2^ = 0.70), and a significant CV-ANOVA (*p*-value = 0.011).

**FIGURE 3:**
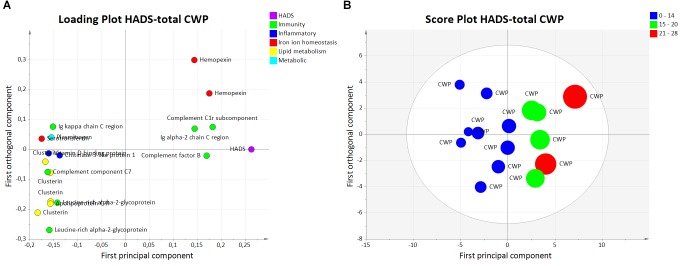
Orthogonal partial least squares regression analysis model HADS-total in the CWP. **(A)** Loading plot corresponding to proteins with a VIP value >1.0 associated to psychological distress are illustrated. The proteins with strongest association to psychological distress are the immunity proteins complement factor B and complement C1r subcomponent and the iron ion homeostasis protein hemopexin. **(B)** Score plot showing a within group separation among CWP based on reported HADS-total score. The plot shows that CWP patients are grouped as normal (0–14), mild (15–20), and moderate (21–28) psychological distress. CWP, chronic widespread pain; HADS, hospital anxiety and depression scale; VIP, variable influence on projection; OPLS, orthogonal partial least squares regression analysis.

A total of 18 proteins had a VIP value >1.0 and were considered significant regressors for HADS-total (*Y*-variable) in the CWP group (Figure 3A). In the score plot of HADS_CWP_, a within group separation is seen among CWP based on the reported HADS-total score (Figure 3B), representing normal (0–14), mild (15–20), and moderate (21–28) psychological distress.

Among the important proteins, the majority of the proteins belonged to immunity processes. Other proteins belonged to inflammatory processes, iron ion homeostasis, lipid metabolism, and metabolic processes (Table 3).

**Table 3:** Orthogonal partial least squares regression analysis model of CWP HADS-total.

Spot number	Protein Name	Accession number	Biological process	Experimental MW (kDa)/p*I*	VIP	p(corr)	OD CON (Mean ± SD)	OD CWP (Mean ± SD)	OD quotient mean	Alteration CWP vs. CON
**1104**	**Clusterin**	**P10909**	**Lipid metabolism**	**44 / 5.03**	**1.36**	−**0.71**	**1649** ± **1628**	**1190** ± **912**	**0.72**	**↓**
**4604**	**Hemopexin**	**P02790**	**Iron ion homeostasis**	**105 / 5.69**	**1.29**	**0.68**	**13505** ± **2499**	**13650** ± **3143**	**1.01**	**↑**
**7901**	**Complement factor B**	**P00751**	**Immunity**	**140 / 6.29**	**1.28**	**0.66**	**1018** ± **515**	**1216** ± **395**	**1.19**	**↑**
**5819**	**Complement C1r subcomponent**	**P00736**	**Immunity**	**137 / 5.66**	**1.27**	**0.71**	**81** ± **110**	**241** ± **155**	**2.98**	**↑**
**3214**	**Clusterin**	**P10909**	**Lipid metabolism**	**42 / 5.26**	**1.23**	−**0.61**	**355** ± **312**	**175** ± **135**	**0.49**	**↓**
6839	Serotransferrin	P02787	Iron ion homeostasis	130 / 6.13	1.21	−0.68	979 ± 458	1078 ± 460	1.10	**↑**
6845	Complement component C7	P10643	Immunity	148 / 6.11	1.21	−0.63	208 ± 277	256 ± 197	1.23	**↑**
1414	Leucine-rich alpha-2-glycoprotein	P02750	Immunity	67 / 4.88	1.14	−0.62	390 ± 202	371 ± 322	0.95	**↓**
1051	Apolipoprotein C-II	P02655	Lipid metabolism	7 / 4.40	1.14	−0.61	483 ± 496	882 ± 893	1.83	**↑**
7819	Plasminogen	P00747	Metabolic	143 / 6.73	1.13	−0.60	325 ± 224	524 ± 280	1.61	**↑**
114	Clusterin	P10909	Lipid metabolism	46 / 4.99	1.12	−0.61	1879 ± 1725	1621 ± 921	0.86	**↓**
1113	Clusterin	P10909	Lipid metabolism	45 / 5.17	1.11	−0.65	1501 ± 1437	1545 ± 1275	1.03	**↑**
1416	Leucine-rich alpha-2-glycoprotein	P02750	Immunity	69 / 4.70	1.11	−0.55	248 ± 217	299 ± 443	1.21	**↑**
7120^∗^	Ig kappa chain C region	P01834	Immunity	28 / 6.29	1.08	−0.59	7997 ± 4977	6563 ± 6880	0.82	**↓**
3408	Vitamin D-binding protein	P02774	Inflammatory	82 / 5.45	1.05	−0.62	404 ± 270	530 ± 286	1.31	**↑**
8306	Chitinase-3-like protein 1	P36222	Inflammatory	54 / 7.11	1.05	−0.53	288 ± 552	276 ± 704	0.96	**↓**
4503	Ig alpha-2 chain C region	P01877	Immunity	90 / 5.66	1.05	0.56	10969 ± 4475	6430 ± 3878	0.59	**↓**
6608	Hemopexin	P02790	Iron ion homeostasis	98 / 5.83	1.03	0.56	1991 ± 657	2157 ± 555	1.08	**↑**

*Proteins with highest VIP values are marked in bold. ^∗^Shared proteoforms with OPLS model of Age_CON_. HADS, hospital anxiety and depression scale; SD, standard deviation; CON, control; CWP, chronic widespread pain; OD, optical density; MW, molecular weight; pI, isoelectric point; VIP, variable influence on projection; OPLS, orthogonal partial least squares regression analysis.*

The five proteins with highest VIP value (VIP > 1.23) were one upregulated proteoform of complement C1r subcomponent (immunity process), complement factor B (immunity process), and hemopexin (iron ion homeostasis process) and two down regulated proteoforms of clusterin (lipid metabolism process) (Table 3). These proteoforms are shown on the 2-DE gel (marked in red) with quantified intensity from individual samples in each group (Figure 2). The spot numbers correspond to spot numbers in Table 3.

#### CON

The OPLS model for CON of HADS-total (HADS_CON_) had one predictive component and one orthogonal component with good sensitivity (*R*^2^ = 0.84), moderate predictivity (*Q*^2^ = 0.48), and a significant CV-ANOVA (*p*-value = 0.016). A total of 12 proteins had a VIP value >1.0 and were considered significant important regressors for HADS (*Y*-variable) in the CON group. The identified proteins belonged to metabolic, immunity, inflammatory and unknown function, where majority of the proteins were metabolic proteins. For more details, see Supplementary Figure S2 and Supplementary Table S2.

### OPLS Models of BMI in CON and CWP

#### CON

To evaluate the correlation of BMI and expressed plasma proteins, an OPLS model of BMI in the CON group (BMI_CON_) was analyzed. The OPLS model for BMI_CON_ consisted of one predictive and one orthogonal component with good sensitivity (*R*^2^ = 0.85), lower predictivity (*Q*^2^ = 0.42), and a significant CV-ANOVA (*p*-value = 0.038).

**FIGURE 4:**
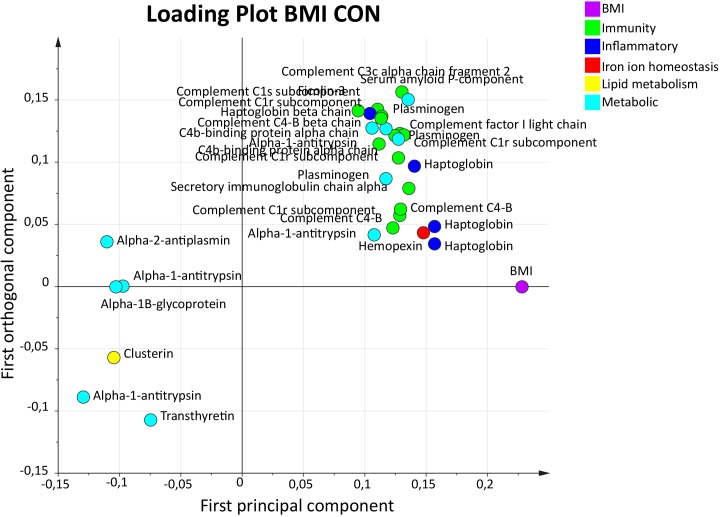
Orthogonal partial least squares regression analysis model BMI in CON. Loading plot illustrating proteins with a VIP value >1.0 associated with BMI. The majority of the proteins belonged to immunity and metabolic responses. Several inflammatory proteins were also present. OPLS, orthogonal partial least squares regression analysis; VIP, variable influence on projection; CON, control; BMI, body mass index.

A total of 31 proteins had a VIP value >1.0 and were considered significant important regressors for BMI (*Y*-variable) in the CON group (Figure 4). The majority of the proteins belonged to immunity and metabolic processes.

The proteins with highest VIP values (VIP > 1.40) were two upregulated proteoforms of haptoglobin (inflammatory process) and one proteoform each of alpha-1-antitrypsin, plasminogen, and serum amyloid P-component (metabolic processes) (Table 4).

**Table 4:** Orthogonal partial least squares regression analysis model of CON and BMI.

Spot number	Protein name	Accession number	Biological process	Experimental MW (kDa)/p*I*	VIP	p(corr)	OD CON (Mean ± SD)	OD CWP (Mean ± SD)	OD quotient mean	Alteration CWP vs. CON
**4119**	**Serum amyloid P-component**	**P02743**	**Metabolic**	**27 / 5.63**	**1.48**	**0.64**	**950** ± **974**	**1474** ± **1330**	**1.55**	**↑**
**4104**	**Haptoglobin**	**P00738**	**Inflammatory**	**53 / 5.74**	**1.45**	**0.75**	**2265** ± **1193**	**3233** ± **1194**	**1.43**	**↑**
**3420**	**Alpha-1- antitrypsin**	**P01009**	**Metabolic**	**71 / 5.26**	**1.44**	−**0.62**	**10036** ± **6237**	**12878** ± **9795**	**1.28**	**↑**
**1204**	**Haptoglobin**	**P00738**	**Inflammatory**	**62 / 5.26**	**1.42**	**0.67**	**10232** ± **6356**	**12766** ± **7283**	**1.25**	**↑**
**8910**	**Plasminogen**	**P00747**	**Metabolic**	**151 / 6.73**	**1.40**	**0.61**	**463** ± **237**	**752** ± **341**	**1.62**	**↑**
4316	Haptoglobin	P00738	Inflammatory	61 / 5.63	1.40	0.75	12243 ± 5754	12341 ± 7702	1.01	**↑**
1111	Complement factor I light chain	P05156	Immunity	48 / 5.22	1.33	0.61	293 ± 273	786 ± 333	2.68	**↑**
5818	Complement C1r subcomponent	P00736	Immunity	137 / 5.63	1.33	0.54	59 ± 94	222 ± 184	3.76	**↑**
145	Complement C3c alpha chain fragment 2	P01024	Immunity	50 / 5.03	1.28	0.62	360 ± 299	869 ± 312	2.41	**↑**
5723	C4b-binding protein alpha chain	P04003	Immunity	116 / 5.80	1.26	0.59	296 ± 422	545 ± 425	1.84	**↑**
5825	Complement C1r subcomponent	P00736	Immunity	130 / 5.66	1.25	0.61	246 ± 144	490 ± 192	1.99	**↑**
8901	Plasminogen	P00747	Metabolic	143 / 7.02	1.24	0.56	796 ± 561	1000 ± 438	1.26	**↑**
3709	Alpha-1B -glycoprotein	P04217	Metabolic	111 / 5.30	1.23	−0.49	600 ± 316	508 ± 301	0.85	**↓**
4808	Secretory immunoglobulin chain alpha	P99003	Immunity	125 / 5.54	1.22	0.65	96 ± 110	88 ± 91	0.92	**↓**
5833	Hemopexin	P02790	Iron ion homeostasis	169 / 5.69	1.21	0.70	78 ± 54	105 ± 104	1.35	**↑**
8101	Complement C4-B	P0C0L5	Immunity	45 / 6.35	1.20	0.61	2511 ± 1437	3344 ± 1364	1.33	**↑**
2116	Transthyretin	P02766	Iron ion homeostasis	13 / 4.75	1.20	−0.36	3497 ± 2539	1265 ± 1382	0.36	**↓**
4809	Complement C1r subcomponent	P00736	Immunity	132 / 5.58	1.19	0.63	158 ± 101	238 ± 155	1.51	**↑**
8823	Plasminogen	P00747	Metabolic	151 / 6.91	1.17	0.56	319 ± 211	652 ± 318	2.04	**↑**
2202	Alpha-1 -antitrypsin	P01009	Metabolic	69 / 5.36	1.15	−0.46	3372 ± 1579	2829 ± 2183	0.84	**↓**
2903	Alpha-1 -antitrypsin	P01009	Metabolic	151 / 5.32	1.12	0.50	57 ± 111	70 ± 47	1.23	**↑**
6713	C4b-binding protein alpha chain	P04003	Immunity	116 / 5.88	1.11	0.53	212 ± 250	438 ± 378	2.07	**↑**
9522	Complement C4-B beta chain	P0C0L5	Immunity	90 / 8.65	1.10	0.54	284 ± 484	584 ± 860	2.06	**↑**
1806	Complement C1s subcomponent	P09871	Immunity	127 / 5.09	1.09	0.53	259 ± 355	626 ± 359	2.42	**↑**
6324	Ficolin-3	O75636	Immunity	47 / 6.00	1.07	0.45	313 ± 271	617 ± 338	1.97	**↑**
3606	Alpha-2 -antiplasmin	P08697	Metabolic	109 / 5.38	1.06	−0.53	187 ± 138	168 ± 211	0.90	**↓**
5824	Complement C1r subcomponent	P00736	Immunity	135 / 5.69	1.05	0.59	162 ± 94	278 ± 129	1.72	**↑**
3809	Alpha-1 -antitrypsin	P01009	Metabolic	148 / 5.22	1.05	0.51	116 ± 161	96 ± 109	0.83	**↓**
3209	Haptoglobin beta chain	P00738	Inflammatory	48 / 5.36	1.03	0.50	210 ± 171	393 ± 186	1.87	**↑**
7216	Complement C4-B	P0C0L5	Immunity	46 / 6.13	1.02	0.61	865 ± 723	1001 ± 920	1.16	**↑**
131	Clusterin	P10909	Lipid metabolism	47 / 4.99	1.02	−0.50	1050 ± 539	1147 ± 806	1.09	**↑**

*31 proteins had a VIP > 1. The proteins with highest VIP belongs to metabolic and inflammatory processes (marked in bold) and was considered as most important for the model. BMI, body mass index; SD, standard deviation; CON, control; CWP, chronic widespread pain; OD, optical density; MW, molecular weight; pI, isoelectric point; VIP, variable influence on projection; OPLS, orthogonal partial least squares regression analysis.*

#### CWP

The OPLS model of BMI for CWP (BMI_CWP_) consisted of one predictive and one orthogonal component with high sensitivity (*R*^2^ = 0.92), predictivity (*Q*^2^ = 0.70), and significant CV-ANOVA (*p*-value = 0.011). A total of 21 proteins had a VIP value >1.0 and were considered significant important regressors for BMI (*Y*-variable) in the CWP group. The identified proteins belonged to immunity, lipid metabolism, and metabolic processes. For more details, see Supplementary Figure S3 and Supplementary Table S3.

### OPLS Models of Age in CON and CWP

#### CON

**FIGURE 5:**
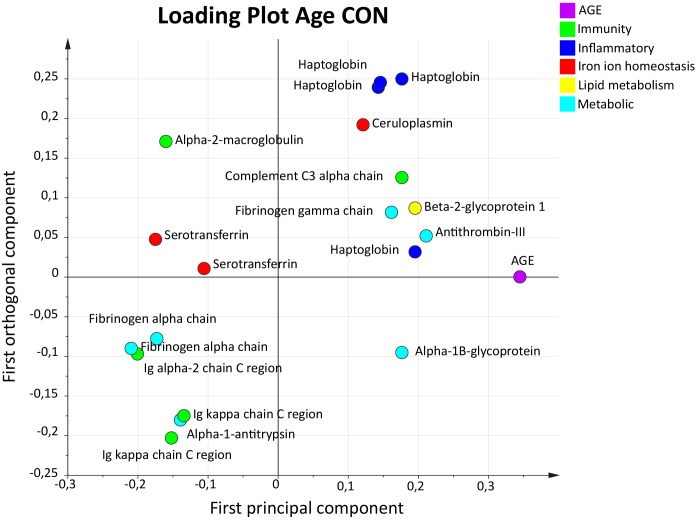
Orthogonal partial least squares regression analysis model of age in CON. Loading plot illustrating proteins with a VIP value >1.0. A variety of different protein groups were present, and proteins with highest VIP value and strongest association to age were one proteoform of haptoglobin and antithrombin-III. OPLS, orthogonal partial least squares regression analysis; VIP, variable influence on projection; CON, control; BMI, body mass index.

To evaluate the influence of age on the expressed plasma proteins, an OPLS model of age in CON (Age_CON_) was analyzed. The OPLS model Age_CON_ had one predictive and one orthogonal component and showed high sensitivity (*R*^2^ = 0.89), predictivity (*Q*^2^ = 0.76), and a significant CV-ANOVA (*p*-value < 0.001). A total of 19 proteins had a VIP value >1.0 and were considered significant important regressors for age (*Y*-variable) in the CON group (Figure 5). Proteins belonging to immunity, inflammatory, iron ion homeostasis, lipid metabolism, and metabolic processes were present.

The five proteins with highest VIP values (VIP > 1.28) were two upregulated proteoforms of fibrinogen alpha chain (metabolic process) and one proteoform of haptoglobin (inflammatory process). One proteoform of Ig alpha-2 chain C region (immunity process) and antithrombin-III (metabolic process) was downregulated (Table 5).

**Table 5:** Orthogonal partial least squares regression analysis model of CON and Age.

Spot number	Protein Name	Accession number	Biological process	Experimental MW (kDa)/p*I*	VIP	p(corr)	OD CON (Mean ± SD)	OD CWP (Mean ± SD)	OD quotient mean	Alteration CWP vs. CON
**6505**	**Ig alpha-2 chain C region**	**P01877**	**Immunity**	**94 / 6.02**	**1.53**	−**0.62**	**2614** ± **1651**	**1772** ± **1267**	**0.68**	**↓**
**3406**	**Antithrombin-III**	**P01008**	**Metabolic**	**76 / 5.54**	**1.52**	**0.65**	**1243** ± **671**	**973** ± **734**	**0.78**	**↓**
**8618**	**Fibrinogen alpha chain**	**P02671**	**Metabolic**	**105 / 7.47**	**1.51**	−**0.65**	**2045** ± **808**	**2365** ± **1147**	**1.16**	**↑**
**5319**	**Haptoglobin**	**P00738**	**Inflammatory**	**54 / 5.88**	**1.28**	**0.60**	**388** ± **232**	**456** ± **539**	**1.18**	**↑**
**8719**	**Fibrinogen alpha chain**	**P02671**	**Metabolic**	**107 / 7.11**	**1.28**	−**0.53**	**3726** ± **1058**	**4040** ± **1837**	**1.08**	**↑**
6407	Beta-2-glycoprotein 1	P02749	Lipid metabolism	81 / 6.00	1.27	0.60	567 ± 125	651 ± 299	1.15	**↑**
7120	Ig kappa chain C region	P01834	Immunity	28 / 6.29	1.23	−0.47	7997 ± 4977	6563 ± 6880	0.82	**↓**
4316	Haptoglobin	P00738	Inflammatory	61 / 5.47	1.20	0.54	12243 ± 5754	12341 ± 7702	1.01	**↑**
2601	Alpha-1B -glycoprotein	P04217	Metabolic	107 / 5.36	1.19	0.54	3245 ± 838	3063 ± 434	0.94	**↓**
7743	Serotransferrin	P02787	Iron ion homeostasis	120 / 6.29	1.18	−0.54	32454 ± 8480	26409 ± 6738	0.81	**↓**
6114	Ig kappa chain C region	P01834	Immunity	28 / 6.05	1.12	−0.41	1733 ± 2089	640 ± 883	0.37	**↓**
6840	Complement C3 alpha chain	P01024	Immunity	157 / 6.08	1.09	0.54	386 ± 169	614 ± 904	1.59	**↑**
5821	Alpha-2 -macroglobulin	P01023	Immunity	176 / 5.71	1.08	−0.49	407 ± 228	579 ± 284	1.42	**↑**
3105	Haptoglobin	P00738	Inflammatory	54 / 5.47	1.05	0.45	2076 ± 1223	2770 ± 1183	1.33	**↑**
2202	Alpha-1 -antitrypsin	P01009	Metabolic	69 / 5.36	1.04	−0.43	3372 ± 1579	2829 ± 2183	0.84	**↓**
6731	Serotransferrin	P02787	Iron ion homeostasis	130 / 6.25	1.03	−0.32	2008 ± 714	1798 ± 710	0.90	**↓**
4833	Ceruloplasmin	P00450	Iron ion homeostasis	163 / 5.51	1.02	0.37	81 ± 109	219 ± 160	2.70	**↑**
2203	Haptoglobin	P00738	Inflammatory	61 / 5.36	1.02	0.44	12006 ± 6246	16032 ± 7681	1.34	**↑**
4301	Fibrinogen gamma chain	P02679	Metabolic	72 / 5.47	1.00	0.50	4565 ± 2265	6308 ± 3044	1.38	**↑**

*19 proteins had a VIP > 1. The 5 proteins with highest VIP values (bold marking) is considered as most important regressor for the model and belonged to immunity, inflammatory and metabolic processes. SD, standard deviation; CON, control; CWP, chronic widespread pain; OD, optical density; MW, molecular weight; pI, isoelectric point; VIP, variable influence on projection; OPLS, orthogonal partial least squares regression analysis.*

#### CWP

The OPLS model of Age in CWP (Age_CWP_) had one predictive and one orthogonal component and showed high sensitivity (*R*^2^ = 0.94), predictivity (*Q*^2^ = 0.77), and a significant CV-ANOVA (*p*-value = 0.003). A total of 12 proteins had a VIP value >1.0 and were considered significant important regressors for age (*Y*-variable) in the CWP group. Proteins belonging to immunity, iron ion homeostasis, lipid metabolism, and metabolic processes were present. For more details, see Supplementary Figure S4 and Supplementary Table S4.

### Shared Proteoforms – Compensating for Possible Cofounding Effects of Age and BMI

To avoid possibly confounding effects of age and BMI on NRS and HADS-total, we compared the models of age and BMI in CON with the models of NRS and HADS-total in CWP. Shared proteoforms were eliminated from the models of NRS_CWP_ and HADS_CWP_, and the models were recalculated.

In the OPLS models of BMI_CON_ and Age_CON_, four proteoforms with a VIP >1.0 (spot number 2116, 2903, 4316, and 4809) were also present in the NRS_CWP_ model. These proteoforms were excluded from the obtained NRS_CWP_ model and the model was re-calculated. The new also significant OPLS model of NRS_CWP_ only had slightly changed parameters (*R*^2^ = 0.96, *Q*^2^ = 0.84, CV-ANOVA; *p* < 0.001) and 16 proteins with a VIP > 1.0 (the same proteins as in first model).

One proteoform (spot number 7120) was shared between the OPLS model of Age_CON_ and HADS_CWP_. No proteoforms were shared between BMI_CON_ and HADS_CWP_. To investigate whether age had a possible cofounding effect on HADS in the models, the shared proteoform was excluded from HADS_CWP_ and a new model was calculated. The new resulting OPLS model for HADS_CWP_ was very similar to the first (*R*^2^ = 0.96, *Q*^2^ = 0.66, CV-ANOVA; *p* < 0.05), with a slightly lower *R*^2^-value and with the same proteins as important regressors with a VIP > 1.0.

### Compensating for Possible Cofounding Effects of HADS-Total Upon NRS_CWP_

After comparing the OPLS models of NRS_CWP_ and HADS_CWP_, one proteoform (spot number 114) was shared and therefore excluded in both models. The new model of NRS_CWP_ (*R*^2^ = 0.96, *Q*^2^ = 0.84, CV-ANOVA; *p* < 0.001) and HADS_CWP_ (*R*^2^ = 0.96, *Q*^2^ = 0.66, CV-ANOVA; *p* < 0.05) had unchanged parameters compared to previous ones.

### Other Shared Proteoforms

In total, 16 proteoforms were shared among other OPLS models. One proteoform of alpha-1-antitrypsin (spot number 2904) was shared between OPLS model NRS_CWP_ and Age_CWP_, and one proteoform of alpha-1-antitrypsin (spot number 2202) was shared between models BMI_CON_ and Age_CON_. Furthermore, two proteoforms of clusterin were shared among Age_CWP_ and BMI_CON_ (spot number 131) and HADS_CWP_ and Age_CWP_ (spot number 1113). Two proteoforms of complement C4-B (spot number 7216 and 8101) was shared between HADS_CON_ and BMI_CON_. Fibrinogen alpha chain (spot number 8719) was shared between Age_CON_ and BMI_CWP_. Fibrinogen gamma chain (spot number 4302 and 4304) was present in both NRS_CWP_ and BMI_CWP_. One proteoform N-acetylmuramoyl-L-alanine amidase (spot number 5721) was present in Age_CWP_ and BMI_CWP_. Three proteoforms of plasminogen were present in several OPLS models: HADS_CWP_ and HADS_CON_ (spot number 7819) and NRS_CWP_ and HADS_CON_ (spot number 8822 and 8909). Two proteoforms of secretory immunoglobulin chain alpha (spot number 4713 and 4807) was present in NRS_CWP_ and BMI_CWP_. Finally, one proteoform of complement factor I light chain (spot number 1111) was present in Age_CWP_ and BMI_CON_ models.

## Discussion

The following are the major results in this present exploratory proteomic study of the plasma in CWP:

• Pain intensity in CWP was associated with several plasma proteins involved in metabolic and immunity processes such as kininogen-1, fibrinogen gamma chain, and ceruloplasmin.
• Psychological distress in CWP was associated with plasma proteins related to immunity response and iron ion metabolism such as complement factor B, complement C1r subcomponent, and hemopexin.
• Proteomics in combination with multivariate statistics can be used to analyze associations between plasma proteins and pain intensity, psychological distress, BMI, and age in CWP and CON.

Overall, investigating the plasma proteome and proteins associated with different clinical measurements, as exemplified in this study, reveals different protein patterns for pain intensity and psychological distress. MVDA is commonly used in proteomic studies to analyze complex biofluids such as urine, plasma, serum, and CSF from CWP (Hadrevi et al., 2015; Backryd et al., 2017a,b; Malatji et al., 2017; Wåhlén et al., 2017). By including the analysis of BMI and age, possible cofounding effects on proteins in each specific model has been evaluated with no major changes in the stability (goodness of fit and model prediction) of pain intensity or psychological distress models. Investigations of the plasma proteome in patients with CWP and/or fibromyalgia have been limited. To the best of our knowledge, no studies have investigated the relationship between pain intensity and psychological distress and the plasma proteome profile.

### Pain Intensity and Plasma Proteins

Pain intensity is an important facet of perception of pain in chronic pain patients. Increased pain intensity is a common feature among CWP patients (Farrar et al., 2001) and, as expected, was relatively high in CWP. The most important proteins that showed the strongest association to pain intensity were upregulated proteoforms of ceruloplasmin, fibrinogen gamma chain, alpha-1B-glycoprotein, and kininogen-1 (Table 2 and Figure 2).

Based on the interpretation of the score plot in combination with the loading plot for NRS_CWP_ (Figures 1A,B), the proteoform of alpha-1B-glycoprotein (one of the top five proteins with a VIP > 1) was associated with lower pain intensity, whereas the other proteins were associated with higher pain intensity (Figure 1A and Table 2). Interestingly, when analyzing NRS_CWP_, the CWP group was divided into subgroups in the score plot according to their reported pain intensity (Figure 1B), reflecting the proteins displayed in each loading plot (Figure 1A). This subdivision within the CWP group could be the result of greater pain intensity being associated with specific metabolic and immunity proteins, which in turn reflects different ongoing protein responses. To test this hypothesis, future studies should analyze CWP patients with severe pain intensity (NRS score >7).

Fibrinogen (comprised of fibrinogen alpha, beta, and gamma chain) and kinogen-1 are known to be involved in different aspects of the blood coagulation cascade (Mosesson, 2005; Sainz et al., 2007; Wu, 2015). Kininogen-1 is primarily involved in the kallikrein-kinin system, and after cleavage one of its products triggers release of bradykinin. Bradykinin is a known mediator of pain (Wang et al., 2005), which further activates the inflammatory response through its indirect production of nitric oxide (NO) and induction of prostaglandins (Cassim et al., 2002). Kininogen-1 and bradykinin expressions were altered in the synovial fluid of rheumatoid arthritis and osteoarthritis patients (Mateos et al., 2012; Wu, 2015). Kiniogen-1 has also been found to be elevated in the plasma of farmers with musculoskeletal disorders (Ghafouri et al., 2016). In this study, both proteoforms of fibrinogen gamma chain and kininogen-1 were upregulated in CWP in the NRS_CWP_ model, which could indicate an increase in inflammatory response.

Ceruloplasmin, an acute-phase protein, is one of the largest transport proteins for copper ions (Hellman et al., 2002). To the best of our knowledge, no direct connection between ceruloplasmin and pain intensity has been reported. Elevated levels of ceruloplasmin have been found in fibromyalgia patients compared to controls, (La Rubia et al., 2013) suggesting altered regulation of copper metabolism may be involved in the pathophysiology of FMS. In our previous plasma proteomic study, we found several proteoforms of ceruloplasmin to be altered and associated with CWP compared to CON (Wåhlén et al., 2017). One of the proteoforms of ceruloplasmin (spot number 3817), found in previous work, was also seen in this study. From the following in-depth analysis, we found that the specific ceruloplasmin proteoform was specifically associated with increased pain intensity.

By studying changes of proteins both systemically and peripherally in the trapezius muscle, the molecular signature and potential biomarkers of pain can be analyzed. For example, from the same cohort different proteoforms of alpha-1-antitrypsin have been found significantly altered in the interstitial fluid of trapezius muscle, a finding confirmed in this study (Olausson et al., 2012). One of the proteoforms of alpha-1-antitrypsin (spot number 2904) was increased and associated with pain intensity; however, the proteoforms from each study had different experimental molecular weights and p*I* (isoelectric point), which could be due to post-translational modifications (PTMs), although this has not been analyzed in detail and needs to be confirmed. The involvement of alpha-1-antitrypsin in fibromyalgia has been reviewed earlier (Blanco et al., 2005), suggesting that it is involved and in favor of an inflammatory response in fibromyalgia patients.

The majority of proteins with a significant association with pain intensity belonged to metabolic processes (according to the UniProt Database). Metabolic proteins have been found to be significantly altered in the trapezius muscle from females with trapezius myalgia and CWP, (Hadrevi et al., 2013; Olausson et al., 2016) suggesting a change in energy metabolism in the muscle. These metabolic proteins seem peripherally and systemically important and involved in mechanisms maintaining CWP.

In summary, pain intensity was associated with plasma proteins involved in metabolic and immunity processes. The most significant proteins were upregulated in CWP. These proteins are known acute-phase protein (ceruloplasmin), which suggests an inflammatory response (fibrinogen and kininogen-1) and changes in energy metabolism.

### Psychological Distress Correlates With Immunity Proteins

The scores from both HADS-anxiety and depression were merged (HADS-total) to get an overall indication of psychological distress. There were significant higher scores in both subscales and HADS-total for CWP compared to CON (Table 1). However, at the group level, both subscales were below the cut-off values of severe symptoms (≥11).

By interpreting the score plot in combination with the loading plot for HADS_CWP_, three of the proteins with highest VIP value were positively associated with psychological distress: complement factor B, complement C1r subcomponent, and hemopexin. Both proteoforms of clusterin were negatively associated with HADS-total in CWP (Figures 3A,B).

Different immune cells such as microglia cells and astrocytes in the brain and macrophages in the periphery can secrete inflammatory substances that in turn can affect peripheral nociceptors in chronic pain (Ji et al., 2013, 2016). These nociceptors can generate action potentials that cause the release of specific ligands in the spinal cord that are ultimately processed by the central nervous system (CNS). They can also receive input and transmission from the CNS out to the periphery, causing local activation of immune cells (Carlton, 2014). The concept of psychoneuroimmunology (PNI) describes a multifaceted interplay between psychological, endocrine, and immune systems, (McCain et al., 2005) which has been described to be involved in fibromyalgia (Menzies et al., 2013). In this study, several of the proteins belonged to immunity process and the CWP group also had an increased psychological distress compared to CON. So far, no studies have investigated the relationship between psychological distress and plasma proteins in CWP. However, increase in pro-inflammatory cytokines and other inflammatory factors in plasma/serum from patients with depression have been reported in several studies (Dowlati et al., 2010; Dahl et al., 2014). Plasma and serum proteomic studies of patients with major depressive disorders have found altered levels of acute phase, complement, and metabolic proteins similar to our study (Lee et al., 2015; Ruland et al., 2016). These studies indicate that changed anxiety or depression status could affect proteins systemically. If this is the case in our study, this might be one explanation for the more immunity and inflammatory proteins seen in CWP. However, the expression of plasma proteins in CWP with severe psychological distress is not known (symptoms that are more related to patients with major depressive disorder), and this should be investigated in future studies.

Complement factor B and complement C1r subcomponent were both upregulated compared to CON and associated with higher HADS-total score in our study (Figures 2, 3A and Table 3). Complement factor B is essential for complement activation through the alternative pathway and activation of adaptive immune response. Complement proteins and coagulation proteins have been reported to be dysregulated in young children who later in life developed psychotic disorders (English et al., 2017).

Clusterin, also known as Apolipoprotein J, is a lipoprotein involved in several different cellular processes as lipid transportation, tissue remodeling, apoptosis and inflammation. Several proteoforms of clusterin were significantly altered and associated with higher psychological distress (Figure 3A and Table 3). Lower levels of serum clusterin has been found associated with pain in patients with hand osteoarthritis compared to healthy controls (Kropackova et al., 2018).

Hemopexin, the largest carrier protein for heme, is classified as an acute phase protein (Tolosano and Altruda, 2002). Free excessive heme (i.e., not bound to hemopexin) can result in production of free radicals and oxidative stress and potentially tissue damage, leading to activation of macrophages, release of cytokines, and induction of inflammation (Ross, 2017). Increased hemopexin levels seen in the HADS_CWP_ model might indicate a protective effect related to a local antioxidant role (Gutteridge and Smith, 1988).

In summary, psychological distress in CWP was associated with plasma proteins related to immunity response and iron ion metabolism. Among the most significant upregulated proteins were complement factor B, complement C1r subcomponent, and hemopexin. Immunity and coagulation proteins play a part in depression; (Song et al., 1994; Lee et al., 2016), however, in this study, CWP at the group level did not exhibit severe symptoms of anxiety and depression (i.e., ≥11) (Zigmond and Snaith, 1983). However, it is plausible that an increase in psychological distress in CWP can affect and even alter immunity proteins even more, which in turn can lead to more intense and prolonged inflammatory response.

### Shared Proteoforms and Post-translational Modifications

One of the advantages of using 2-DE is its ability to detect and investigate a protein’s different proteoforms and its PTMs such as truncation, phosphorylation, and glycosylation. In this study, different proteoforms of the same protein were found differentially expressed in the different MVDA models. For example, four proteoforms of clusterin were differentially expressed – one form was up-regulated and three were down regulated – in the HADS_CWP_ model (Table 3). Differentially charged proteoforms of clusterin in plasma have been reported (Ghafouri et al., 2016). Findings like these are normally missed when using 1D or LC based proteomics. However, the physiological relevance of these different proteoforms in chronic pain have yet to be fully understood. Further studies to characterize the different proteoforms are needed before analysis with immunoassay such as western blot. By using immunoassay it is possible to detect/quantify the total amount of clusterin and to the best of our knowledge there are no commercial available antibodies against the different proteoforms of clusterin.

Glycosylated proteins may have a prolonged half-life time in circulation (Flintegaard et al., 2010). If this is the case for several of the proteins found in this study, an overall increase of these proteins might contribute to a sustained inflammatory response. Furthermore, PTMs of ion channels involved in peripheral sensitization have been well-reviewed by Bhave and Gereau (2004) and Laedermann et al. (2015) PTMs like phosphorylation of the capsaicin receptor (Transient receptor potential vanilloid 1, TRPV1) and sodium, potassium, and calcium channels expressed in primary sensory neurons can alter both the function and expression and thereby affect the transducing capability and excitability of the receptors. Although the proteins found in this study are not covered by these articles, PTMs are suggested to be involved in chronic pain. Therefore, future studies could use proteomics to detect different proteoforms with potential PTMs in different pain conditions of interest.

### Shared Plasma Proteins in NRS_CWP_, HADS_CWP_, and OPLS-DA Model of CWP and CON

In total among all models presented in this study, 21 proteins were seen in our previous study comparing group differences of plasma proteins between CWP and CON using OPLS-DA analysis (Wåhlén et al., 2017). Specifically, six proteoforms (spot number 3817, 4304, 4302, 8822, 2116, and 4810) were shared among NRS_CWP_ and two proteoforms (5819 and 4503) in HADS_CWP_ compared to the OPLS-DA model of CWP and CON. Interestingly, in the NRS_CWP_ model four out of these six proteoforms (spot number 3817, 4302,4304, and 8822) are the proteoforms with highest VIP value (Table 2). These proteins have been identified on both group levels with strong association to CWP and specifically related to pain intensity. In the previous study, transthyretin (spot number 2116) was more associated to CON, which is further shown in this study since it was found in BMI_CON_ (Table 4) and therefore excluded in the NRS_CWP_ analysis. MVDA allows for the analysis of the protein patterns both on the group level, followed by in-depth analysis, and on its association to clinical parameters, which potentially could reflect the ongoing mechanisms involved in CWP.

### Low-Grade Systemic Inflammation in CWP Patients

Using the same cohort, our recent analysis of the inflammatory cytokine/chemokine profile suggested a low-grade inflammation in CWP patients (Gerdle et al., 2017). The CSF proteome was analyzed in an additional cohort and showed that the proteins were involved in the immune system, apoptotic regulations, anti-inflammatory, and anti-oxidative processes, indicating presence of neuro-inflammation in the CNS of the CWP patients (Olausson et al., 2017). These results are in line with the plasma profile seen in this study and were the most discriminant proteins belonging to immunity and metabolic responses highly involved in inflammatory processes.

### Strengths and Limitations

The complexity of plasma sample or other biofluids and its limitation to detect all proteins or other metabolites with one single method remains difficult. However, one advantage with using traditional 2-DE is the interpretation of a protein’s different proteoforms and potential PTMs, which has shown in this study to be of importance since a majority of the significant proteins are expressed as different proteoforms. Furthermore, 2-DE is limited in detecting large hydrophobic proteins (>200 kDa) and small peptides (<10 kDa). A gel’s gradient composition and the resolution of proteins in the first dimension could also affect the number of detected proteins in each gel. In this study, removal of albumin and IgG from plasma was used as pre-treatment of the plasma sample. It is possible that the removal procedure might have eliminated some other candidates that could be of interest. Further studies using fractionation could be used to generate a data set with more protein identifications and improved confidence. The advantage of MVDA is its application on large scale data produced when using proteomics. We have shown that MVDA is highly applicable in the proteomic field of chronic pain both when comparing proteins on a group level and when interpreting clinical parameters and its association with plasma proteins.

The interpretation of the result in this study should be taken with precaution due to its small sample size. In future studies, sample size needs to be increased. However, due to restrict inclusion/exclusion criteria we – in the context of chronic pain conditions – has achieved a relatively homogenous group of CWP patients even though some degree of heterogeneity cannot be excluded. The recruitment of the subjects in the present study took place before the current revised ACR criteria of 2016 were accepted. In future larger studies – during a transition period – it may be an advantage to describe the patients using both the ACR criteria from 1990 and the revised criteria from 2016. Moreover, future studies should include CWP patients with high and low psychological distress as well as pain free subjects with depression and/or anxiety or other psychiatric conditions can be used as positive controls. In CWP, a prominent clinical feature is fatigue and in future studies this symptom needs to be considered. In addition, it is a cross sectional study that analyses the plasma proteome at one specific time. Another aspect worth considering when validating the results is the application of additional proteomic methods such as shot gun proteomics.

## Conclusion

This study suggests that different plasma protein patterns are associated with different pain intensities and psychological distress in CWP. Proteins belonging to the coagulation cascade and immunity processes showed strong association to each clinical outcome. Using the plasma proteome profile of CWP to study potential biomarker candidates provides a snapshot of ongoing systemic mechanisms in CWP. The effect seen systemically might be an effect of local peripheral changes in muscles and/or central changes such as central sensitization. This study suggests a numbers of potential candidates of plasma biomarkers in chronic pain that needs to be verified in different cohorts.

## Author Contributions

KW, BGh, BG, and NG designed the experiments and critically revising the paper and agree to be accountable for all aspects of the work. KW and BGh performed the experiments. KW, BGh, and BGe analyzed the data. KW and BGe wrote the original draft.

## Conflict of Interest Statement

The authors declare that the research was conducted in the absence of any commercial or financial relationships that could be construed as a potential conflict of interest.

## Footnotes

1 www.uniprot.org

## References

[B1] Anderson N. L. Anderson N. G. (2002). The human plasma proteome: history, character, and diagnostic prospects. *Mol. Cell. Proteom.* 1 845–867. 10.1074/mcp.R200007-MCP200 12488461

[B2] Aparicio V. A. Ortega F. B. Carbonell-Baeza A. Gatto-Cardia C. Sjöström M. Ruiz J. R. (2013). Fibromyalgia’s key symptoms in normal-weight, overweight, and obese female patients. *Pain Manag. Nurs.* 14 268–276. 10.1016/j.pmn.2011.06.002 24315250

[B3] Backryd E. Lind A. L. Thulin M. Larsson A. Gerdle B. Gordh T. (2017a). High levels of cerebrospinal fluid chemokines point to the presence of neuroinflammation in peripheral neuropathic pain: a cross-sectional study of 2 cohorts of patients compared with healthy controls. *Pain* 158 2487–2495. 10.1097/j.pain.0000000000001061 28930774 PMC5690569

[B4] Backryd E. Tanum L. Lind A. L. Larsson A. Gordh T. (2017b). Evidence of both systemic inflammation and neuroinflammation in fibromyalgia patients, as assessed by a multiplex protein panel applied to the cerebrospinal fluid and to plasma. *J. Pain Res.* 10 515–525. 10.2147/JPR.S128508 28424559 PMC5344444

[B5] Bazzichi L. Ciregia F. Giusti L. Baldini C. Giannaccini G. Giacomelli C. (2009). Detection of potential markers of primary fibromyalgia syndrome in human saliva. *Proteomics Clin. Appl.* 3 1296–1304. 10.1002/prca.200900076 21136951

[B6] Bergman S. Herrstrom P. Hogstrom K. Petersson I. F. Svensson B. Jacobsson L. T. (2001). Chronic musculoskeletal pain, prevalence rates, and sociodemographic associations in a Swedish population study. *J. Rheumatol.* 28 1369–1377. 11409133

[B7] Bhave G. Gereau R. W. T. (2004). Posttranslational mechanisms of peripheral sensitization. *J. Neurobiol.* 61 88–106. 10.1002/neu.20083 15362155

[B8] Bjelland I. Dahl A. A. Haug T. T. Neckelmann D. (2002). The validity of the hospital anxiety and depression scale. An updated literature review. *J. Psychosom. Res.* 52 69–77. 10.1016/S0022-3999(01)00296-3 11832252

[B9] Blanco L. E. de Serres F. J. Fernandez-Bustillo E. Kassam D. A. Arbesú D. Rodríguez C. (2005). alpha1-Antitrypsin and fibromyalgia: new data in favour of the inflammatory hypothesis of fibromyalgia. *Med. Hypotheses* 64 759–769. 10.1016/j.mehy.2004.10.005 15694694

[B10] Breivik H. Collett B. Ventafridda V. Cohen R. Gallacher D. (2006). Survey of chronic pain in Europe: prevalence, impact on daily life, and treatment. *Eur. J. Pain* 10 287–333. 10.1016/j.ejpain.2005.06.009 16095934

[B11] Cao Z. Tang H. Y. Wang H. Liu Q. Speicher D. W. (2012). Systematic comparison of fractionation methods for in-depth analysis of plasma proteomes. *J. Proteome Res.* 11 3090–3100. 10.1021/pr201068b 22536952 PMC3430803

[B12] Carlton S. M. (2014). Nociceptive primary afferents: they have a mind of their own. *J. Physiol.* 592 3403–3411. 10.1113/jphysiol.2013.269654 24879874 PMC4229338

[B13] Cassim B. Mody G. Bhoola K. (2002). Kallikrein cascade and cytokines in inflamed joints. *Pharmacol. Ther.* 94 1–34. 10.1016/S0163-7258(02)00166-3 12191591

[B14] Cimmino M. A. Ferrone C. Cutolo M. (2011). Epidemiology of chronic musculoskeletal pain. *Best Pract. Res. Clin. Rheumatol.* 25 173–183. 10.1016/j.berh.2010.01.012 22094194

[B15] Culic O. Cordero M. D. Zanic-Grubisic T. Somborac-Baèura A. Puèar L. B. Detel D. (2016). Serum activities of adenosine deaminase, dipeptidyl peptidase IV and prolyl endopeptidase in patients with fibromyalgia: diagnostic implications. *Clin. Rheumatol.* 35 2565–2571. 10.1007/s10067-016-3377-8 27527091

[B16] Dahl J. Ormstad H. Aass H. C. Malt U. F. Bendz L. T. Sandvik L. (2014). The plasma levels of various cytokines are increased during ongoing depression and are reduced to normal levels after recovery. *Psychoneuroendocrinology* 45 77–86. 10.1016/j.psyneuen.2014.03.019 24845179

[B17] Dowlati Y. Herrmann N. Swardfager W. Liu H. Sham L. Reim E. K. (2010). A meta-analysis of cytokines in major depression. *Biol. Psychiatry* 67 446–457. 10.1016/j.biopsych.2009.09.033 20015486

[B18] English J. A. Lopez L. M. O’Gorman A. Föcking M. Hryniewiecka M. Scaife C. (2017). Blood-based protein changes in childhood are associated with increased risk for later psychotic disorder: evidence from a nested case-control study of the ALSPAC longitudinal birth cohort. *Schizophr. Bull.* 44 297–306. 10.1093/schbul/sbx075 29036721 PMC5814944

[B19] Eriksson L. Johansson E. Kettaneh-Wold N. Trygg J. Wikström C. Wold S. (2006). *Multi- and Megavariate Data Analysis; Part I and II*, 2 Edn. Umeå: Umetrics AB.

[B20] Farrar J. T. Young J. P. Jr. LaMoreaux L. Werth J. L. Poole R. M. (2001). Clinical importance of changes in chronic pain intensity measured on an 11-point numerical pain rating scale. *Pain* 94 149–158. 10.1016/S0304-3959(01)00349-9 11690728

[B21] Ferreira-Valente M. A. Pais-Ribeiro J. L. Jensen M. P. (2011). Validity of four pain intensity rating scales. *Pain* 152 2399–2404. 10.1016/j.pain.2011.07.005 21856077

[B22] Flintegaard T. V. Thygesen P. Rahbek-Nielsen H. Levery S. B. Kristensen C. Clausen H. (2010). N-glycosylation increases the circulatory half-life of human growth hormone. *Endocrinology* 151 5326–5336. 10.1210/en.2010-0574 20826563

[B23] Flodin P. Martinsen S. Lofgren M. Bileviciute-Ljungar I. Kosek E. Fransson P. (2014). Fibromyalgia is associated with decreased connectivity between pain- and sensorimotor brain areas. *Brain Connect.* 4 587–594. 10.1089/brain.2014.0274 24998297 PMC4202907

[B24] Gerdle B. Ghafouri B. Ghafouri N. Backryd E. Gordh T. (2017). Signs of ongoing inflammation in female patients with chronic widespread pain: a multivariate, explorative, cross-sectional study of blood samples. *Medicine* 96:e6130. 10.1097/MD.0000000000006130 28248866 PMC5340439

[B25] Gerdle B. Larsson B. Forsberg F. Ghafouri N. Karlsson L. Stensson N. (2014). Chronic widespread pain: increased glutamate and lactate concentrations in the trapezius muscle and plasma. *Clin. J. Pain* 30 409–420. 10.1097/AJP.0b013e31829e9d2a 23887335

[B26] Gerdle B. Soderberg K. Salvador Puigvert L. Rosendal L. Larsson B. (2010). Increased interstitial concentrations of pyruvate and lactate in the trapezius muscle of patients with fibromyalgia: a microdialysis study. *J. Rehabil. Med.* 42 679–687. 10.2340/16501977-0581 20603699

[B27] Geyer P. E. Holdt L. M. Teupser D. Mann M. (2017). Revisiting biomarker discovery by plasma proteomics. *Mol. Syst. Biol.* 13:942. 10.15252/msb.20156297 28951502 PMC5615924

[B28] Ghafouri B. Carlsson A. Holmberg S. Thelin A. Tagesson C. (2016). Biomarkers of systemic inflammation in farmers with musculoskeletal disorders; a plasma proteomic study. *BMC Musculoskelet. Disord.* 17:206. 10.1186/s12891-016-1059-y 27160764 PMC4862124

[B29] Ghafouri N. Ghafouri B. Larsson B. Stensson N. Fowler C. J. Gerdle B. (2013). Palmitoylethanolamide and stearoylethanolamide levels in the interstitium of the trapezius muscle of women with chronic widespread pain and chronic neck-shoulder pain correlate with pain intensity and sensitivity. *Pain* 154 1649–1658. 10.1016/j.pain.2013.05.002 23707281

[B30] Gorg A. Drews O. Luck C. Weiland F. Weiss W. (2009). 2-DE with IPGs. *Electrophoresis* 30(Suppl. 1), S122–S132. 10.1002/elps.200900051 19441019

[B31] Gutteridge J. M. Smith A. (1988). Antioxidant protection by haemopexin of haem-stimulated lipid peroxidation. *Biochem. J.* 256 861–865. 10.1042/bj2560861 3223958 PMC1135495

[B32] Hadrevi J. Bjorklund M. Kosek E. Hällgren S. Antti H. Fahlström M. (2015). Systemic differences in serum metabolome: a cross sectional comparison of women with localised and widespread pain and controls. *Sci. Rep.* 5:15925. 10.1038/srep15925 26522699 PMC4629114

[B33] Hadrevi J. Ghafouri B. Larsson B. Gerdle B. Hellstrom F. (2013). Multivariate modeling of proteins related to trapezius myalgia, a comparative study of female cleaners with or without pain. *PLoS One* 8:e73285. 10.1371/journal.pone.0073285 24023854 PMC3762788

[B34] Hellman N. E. Kono S. Mancini G. M. Hoogeboom A. J. De Jong G. J. Gitlin J. D. (2002). Mechanisms of copper incorporation into human ceruloplasmin. *J. Biol. Chem.* 277 46632–46638. 10.1074/jbc.M206246200 12351628

[B35] Ji R. R. Berta T. Nedergaard M. (2013). Glia and pain: is chronic pain a gliopathy? *Pain* 154(Suppl. 1), S10–S28. 10.1016/j.pain.2013.06.022 23792284 PMC3858488

[B36] Ji R. R. Chamessian A. Zhang Y. Q. (2016). Pain regulation by non-neuronal cells and inflammation. *Science* 354 572–577. 10.1126/science.aaf8924 27811267 PMC5488328

[B37] Kliger M. Stahl S. Haddad M. Suzan E. Adler R. Eisenberg E. (2015). Measuring the intensity of chronic pain: are the visual analogue scale and the verbal rating scale interchangeable? *Pain Pract.* 15 538–547. 10.1111/papr.12216 24735056

[B38] Kropackova T. Sleglova O. Ruzickova O. Vencovsky J. Pavelka K. Senolt L. (2018). Lower serum clusterin levels in patients with erosive hand osteoarthritis are associated with more pain. *BMC Musculoskelet. Disord.* 19:264. 10.1186/s12891-018-2179-3 30053814 PMC6064100

[B39] La Rubia M. Rus A. Molina F. Del Moral M. L. (2013). Is fibromyalgia-related oxidative stress implicated in the decline of physical and mental health status? *Clin. Exp. Rheumatol.* 31(6 Suppl. 79), S121–S127. 24373370

[B40] Laedermann C. J. Abriel H. Decosterd I. (2015). Post-translational modifications of voltage-gated sodium channels in chronic pain syndromes. *Front. Pharmacol.* 6:263. 10.3389/fphar.2015.00263 26594175 PMC4633509

[B41] Lee J. Joo E. J. Lim H. J. Park J. M. Lee K. Y. Park A. (2015). Proteomic analysis of serum from patients with major depressive disorder to compare their depressive and remission statuses. *Psychiatry Investig.* 12 249–259. 10.4306/pi.2015.12.2.249 25866527 PMC4390597

[B42] Lee M. Y. Kim E. Y. Kim S. H. Cho K. C. Ha K. Kim K. P. (2016). Discovery of serum protein biomarkers in drug-free patients with major depressive disorder. *Prog. Neuropsychopharmacol. Biol. Psychiatry* 69 60–68. 10.1016/j.pnpbp.2016.04.009 27105922

[B43] Lisspers J. Nygren A. Soderman E. (1997). Hospital anxiety and depression scale (HAD): some psychometric data for a Swedish sample. *Acta Psychiatr. Scand.* 96 281–286. 10.1111/j.1600-0447.1997.tb10164.x 9350957

[B44] Liu X. D. Zeng B. F. Xu J. G. Zhu H. B. Xia Q. C. (2006). Proteomic analysis of the cerebrospinal fluid of patients with lumbar disk herniation. *Proteomics* 6 1019–1028. 10.1002/pmic.200500247 16372267

[B45] Magdeldin S. Enany S. Yoshida Y. Xu B. Zhang Y. Zureena Z. (2014). Basics and recent advances of two dimensional- polyacrylamide gel electrophoresis. *Clin. Proteomics* 11:16. 10.1186/1559-0275-11-16 24735559 PMC3996944

[B46] Malatji B. G. Meyer H. Mason S. Engelke U. F. H. Wevers R. A. van Reenen M. (2017). A diagnostic biomarker profile for fibromyalgia syndrome based on an NMR metabolomics study of selected patients and controls. *BMC Neurol.* 17:88. 10.1186/s12883-017-0863-9 28490352 PMC5426044

[B47] Mansfield K. E. Sim J. Jordan J. L. Jordan K. P. (2016). A systematic review and meta-analysis of the prevalence of chronic widespread pain in the general population. *Pain* 157 55–64. 10.1097/j.pain.0000000000000314 26270591 PMC4711387

[B48] Mateos J. Lourido L. Fernandez-Puente P. Calamia V. Fernández-López C. Oreiro N. (2012). Differential protein profiling of synovial fluid from rheumatoid arthritis and osteoarthritis patients using LC-MALDI TOF/TOF. *J. Proteomics* 75 2869–2878. 10.1016/j.jprot.2011.12.042 22245418

[B49] McCain N. L. Gray D. P. Walter J. M. Robins J. (2005). Implementing a comprehensive approach to the study of health dynamics using the psychoneuroimmunology paradigm. *ANS Adv. Nurs. Sci.* 28 320–332. 10.1097/00012272-200510000-00004 16292018 PMC2213424

[B50] Menzies V. Lyon D. E. Elswick R. K. Jr. Montpetit A. J. McCain N. L. (2013). Psychoneuroimmunological relationships in women with fibromyalgia. *Biol. Res. Nurs.* 15 219–225. 10.1177/1099800411424204 22174319 PMC3722552

[B51] Mosesson M. W. (2005). Fibrinogen and fibrin structure and functions. *J. Thromb. Haemost.* 3 1894–1904. 10.1111/j.1538-7836.2005.01365.x 16102057

[B52] Neumann L. Lerner E. Glazer Y. Bolotin A. Shefer A. Buskila D. (2008). A cross-sectional study of the relationship between body mass index and clinical characteristics, tenderness measures, quality of life, and physical functioning in fibromyalgia patients. *Clin. Rheumatol.* 27 1543–1547. 10.1007/s10067-008-0966-1 18622575

[B53] Olausson P. Gerdle B. Ghafouri N. Larsson B. Ghafouri B. (2012). Identification of proteins from interstitium of trapezius muscle in women with chronic myalgia using microdialysis in combination with proteomics. *PLoS One* 7:e52560. 10.1371/journal.pone.0052560 23300707 PMC3531451

[B54] Olausson P. Gerdle B. Ghafouri N. Sjostrom D. Blixt E. Ghafouri B. (2015). Protein alterations in women with chronic widespread pain–An explorative proteomic study of the trapezius muscle. *Sci. Rep.* 5:11894. 10.1038/srep11894 26150212 PMC4493691

[B55] Olausson P. Ghafouri B. Backryd E. Gerdle B. (2017). Clear differences in cerebrospinal fluid proteome between women with chronic widespread pain and healthy women - a multivariate explorative cross-sectional study. *J. Pain Res.* 10 575–590. 10.2147/JPR.S125667 28331360 PMC5356922

[B56] Olausson P. Ghafouri B. Ghafouri N. Gerdle B. (2016). Specific proteins of the trapezius muscle correlate with pain intensity and sensitivity - an explorative multivariate proteomic study of the trapezius muscle in women with chronic widespread pain. *J. Pain Res.* 9 345–356. 10.2147/JPR.S102275 27330327 PMC4898258

[B57] Perez de Heredia-Torres M. Huertas-Hoyas E. Maximo-Bocanegra N. Palacios-Cena D. Fernandez-De-Las-Penas C. (2016). Cognitive performance in women with fibromyalgia: a case-control study. *Aust. Occup. Ther. J.* 63 329–337. 10.1111/1440-1630.12292 27059423

[B58] Ross A. C. (2017). Impact of chronic and acute inflammation on extra- and intracellular iron homeostasis. *Am. J. Clin. Nutr.* 106(Suppl. 6), 1581S–1587S. 10.3945/ajcn.117.155838 29070546 PMC5701715

[B59] Ruland T. Chan M. K. Stocki P. Grosse L. Rothermundt M. Cooper J. D. (2016). Molecular serum signature of treatment resistant depression. *Psychopharmacology* 233 3051–3059. 10.1007/s00213-016-4348-0 27325393

[B60] Rus A. Molina F. Gasso M. Camacho M. V. Peinado M. A. del Moral M. L. (2016). Nitric oxide, inflammation, lipid profile, and cortisol in normal- and overweight women with fibromyalgia. *Biol. Res. Nurs.* 18 138–146. 10.1177/1099800415591035 26134428

[B61] Sainz I. M. Pixley R. A. Colman R. W. (2007). Fifty years of research on the plasma kallikrein-kinin system: from protein structure and function to cell biology and in-vivo pathophysiology. *Thromb. Haemost.* 98 77–83. 10.1160/TH07-04-0250 17597995

[B62] Song C. Dinan T. Leonard B. E. (1994). Changes in immunoglobulin, complement and acute phase protein levels in the depressed patients and normal controls. *J. Affect. Disord.* 30 283–288. 10.1016/0165-0327(94)90135-X 7516941

[B63] Staud R. Nagel S. Robinson M. E. Price D. D. (2009). Enhanced central pain processing of fibromyalgia patients is maintained by muscle afferent input: a randomized, double-blind, placebo-controlled study. *Pain* 145 96–104. 10.1016/j.pain.2009.05.020 19540671 PMC2751583

[B64] Staud R. Vierck C. J. Cannon R. L. Mauderli A. P. Price D. D. (2001). Abnormal sensitization and temporal summation of second pain (wind-up) in patients with fibromyalgia syndrome. *Pain* 91 165–175. 10.1016/S0304-3959(00)00432-2 11240089

[B65] Tolosano E. Altruda F. (2002). Hemopexin: structure, function, and regulation. *DNA Cell Biol.* 21 297–306. 10.1089/104454902753759717 12042069

[B66] Wåhlén K. Olausson P. Carlsson A. Ghafouri N. Gerdle B. Ghafouri B. (2017). Systemic alterations in plasma proteins from women with chronic widespread pain compared to healthy controls: a proteomic study. *J. Pain Res.* 10 797–809. 10.2147/JPR.S128597 28435317 PMC5388344

[B67] Wang H. Kohno T. Amaya F. Brenner G. J. Ito N. Allchorne A. (2005). Bradykinin produces pain hypersensitivity by potentiating spinal cord glutamatergic synaptic transmission. *J. Neurosci.* 25 7986–7992. 10.1523/JNEUROSCI.2393-05.2005 16135755 PMC6725443

[B68] Westermeier R. Gorg A. (2011). Two-dimensional electrophoresis in proteomics. *Methods Biochem. Anal.* 54 411–439. 10.1002/9780470939932.ch17 21954788

[B69] Wheelock A. M. Wheelock C. E. (2013). Trials and tribulations of ’omics data analysis: assessing quality of SIMCA-based multivariate models using examples from pulmonary medicine. *Mol. Biosyst.* 9 2589–2596. 10.1039/c3mb70194h 23999822

[B70] Wolfe F. Smythe H. A. Yunus M. B. Bennett R. M. Bombardier C. Goldenberg D. L. (1990). The American college of rheumatology criteria for the classification of fibromyalgia. Report of the multicenter criteria committee. *Arthritis Rheum.* 1990 160–172. 10.1002/art.1780330203 2306288

[B71] Wu Y. (2015). Contact pathway of coagulation and inflammation. *Thromb. J.* 13:17. 10.1186/s12959-015-0048-y 25949215 PMC4421925

[B72] Xiao Y. Haynes W. L. Michalek J. E. Russell I. J. (2013). Elevated serum high-sensitivity C-reactive protein levels in fibromyalgia syndrome patients correlate with body mass index, interleukin-6, interleukin-8, erythrocyte sedimentation rate. *Rheumatol. Int.* 33 1259–1264. 10.1007/s00296-012-2538-6 23124693

[B73] Zanette S. A. Dussan-Sarria J. A. Souza A. Deitos A. Torres I. L. S. Caumo W. (2014). Higher serum S100B and BDNF levels are correlated with a lower pressure-pain threshold in fibromyalgia. *Mol. Pain* 10:46. 10.1186/1744-8069-10-46 25005881 PMC4094546

[B74] Zhang Y. G. Jiang R. Q. Guo T. M. Wu S. X. Ma W. J. (2014). MALDI-TOF-MS serum protein profiling for developing diagnostic models and identifying serum markers for discogenic low back pain. *BMC Musculoskelet. Disord.* 15:193. 10.1186/1471-2474-15-193 24889399 PMC4061098

[B75] Zigmond A. S. Snaith R. P. (1983). The hospital anxiety and depression scale. *Acta Psychiatr. Scand.* 67 361–370. 10.1111/j.1600-0447.1983.tb09716.x 6880820

