# Supplementary material for: Plasma Protein Pattern Correlates With Pain Intensity and Psychological Distress in Women With Chronic Widespread Pain
Source: Front Psychol. 2018 Nov 29;9:2400. doi: 10.3389/fpsyg.2018.02400 (PMC6281753; doi:10.3389/fpsyg.2018.02400)

Supplementary Figures

Supplementary Figure S1. Plasma protein pattern and significant proteoforms from all MVDA models. Marked protein spot numbers corresponds to equal number in supplementary table S1. p*I*: isoelectric point, MW: molecular weight

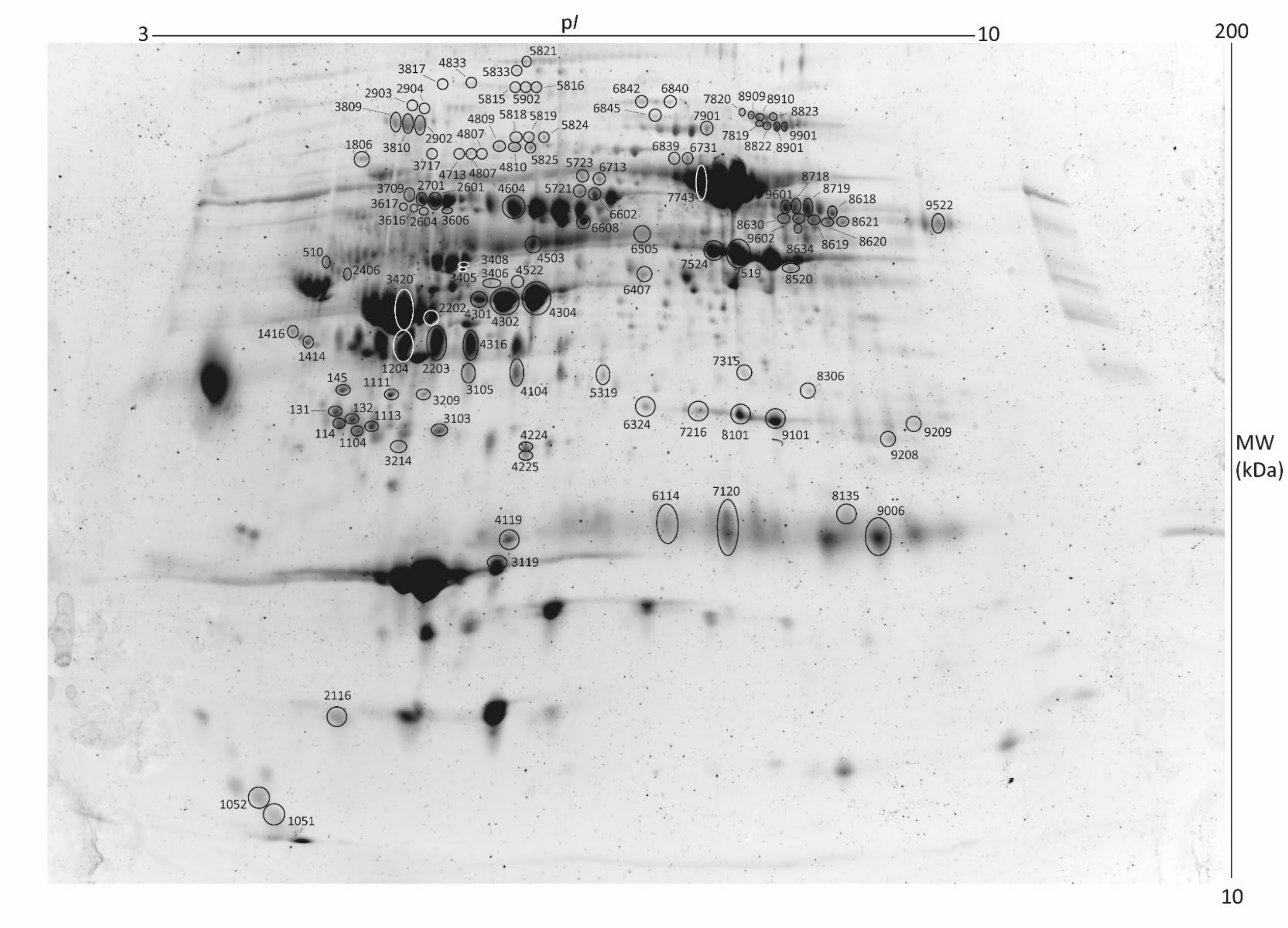

Supplementary Figure S2. **OPLS model of HADS-total in CON group.** Loading plot illustrating proteins with a VIP value > 1.0 associated with HADS-total in CON. All proteins belonged to either immunity or metabolic processes. The ones with highest VIP value were different proteoforms of plasminogen and alpha-2-antiplasmin, secretory immunoglobulin chain alpha.

OPLS; orthogonal partial least squares regression analysis, HADS; hospital anxiety and depression scale VIP; variable influence on projection, CON; control

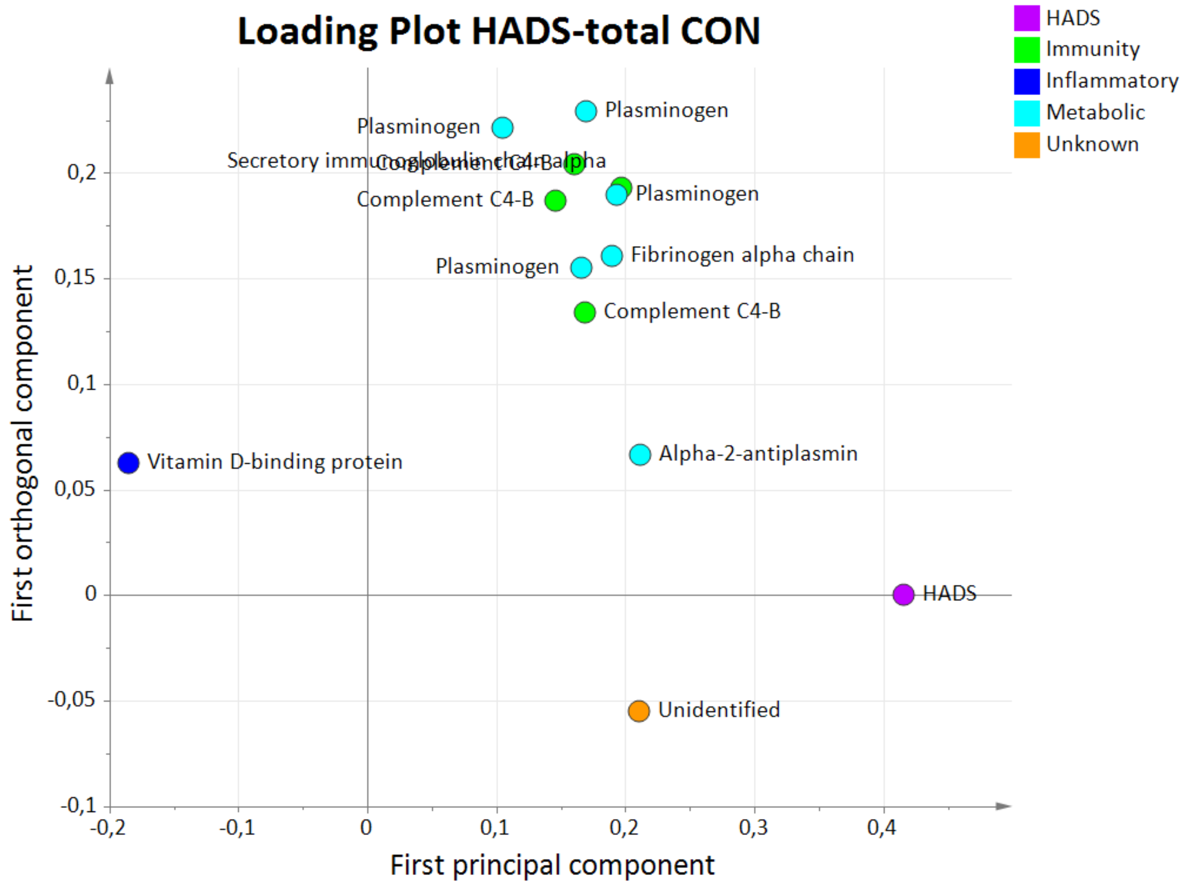

Supplementary Figure S3. **OPLS model of BMI in CWP group.** Loading plot illustrating proteins with a VIP value > 1.0 associated with BMI in CWP. Majority of the proteins belonged to metabolic processes, and the ones with highest VIP value were different proteoforms of fibrinogen alpha and gamma chain.

OPLS; orthogonal partial least squares regression analysis, BMI; body mass index, VIP; variable influence on projection, CWP; chronic widespread pain

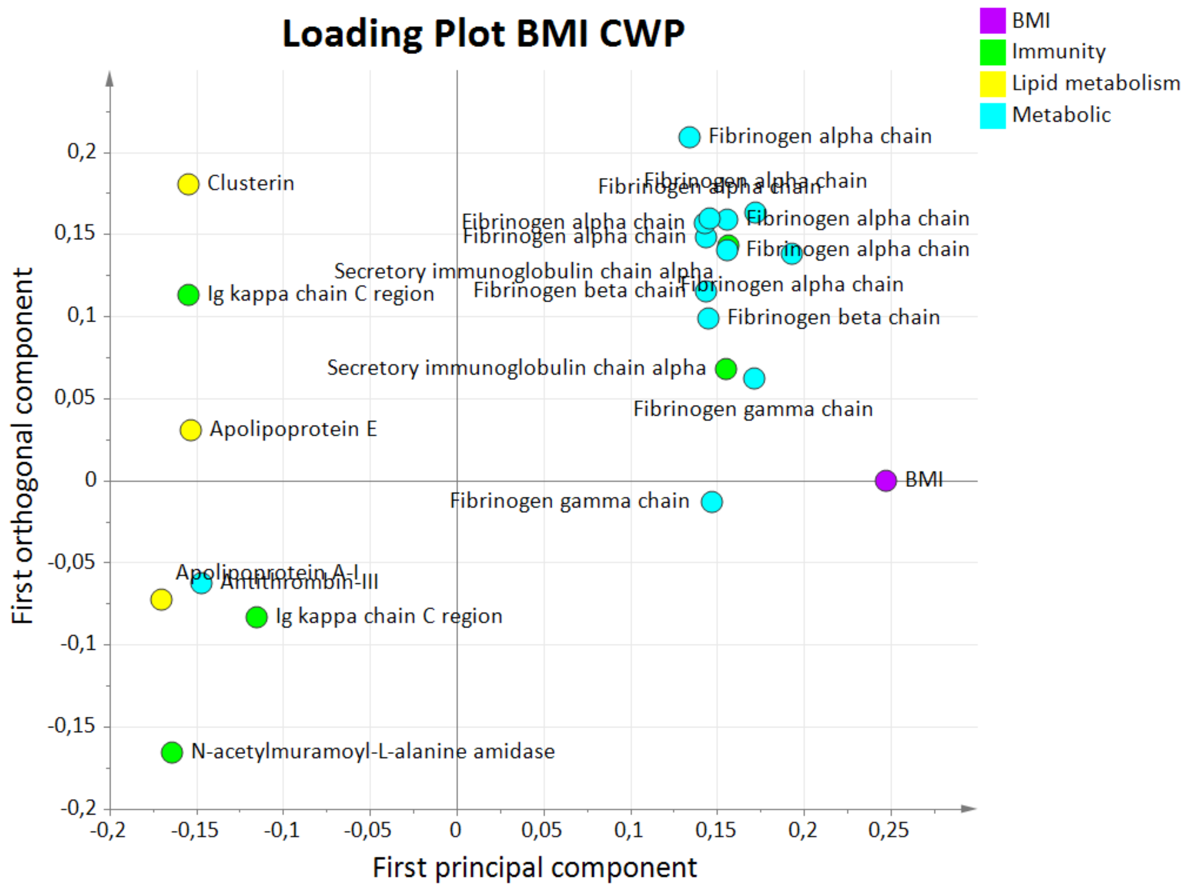

Supplementary Figure S4. **OPLS model of Age in CWP group.** Loading plot illustrating proteins with a VIP value > 1.0 associated with age in CWP. The proteins with highest VIP were several proteoforms of clusterin and apolipoprotein E, belonging to lipid metabolism.

OPLS; orthogonal partial least squares regression analysis, VIP; variable influence on projection, CWP; chronic widespread pain

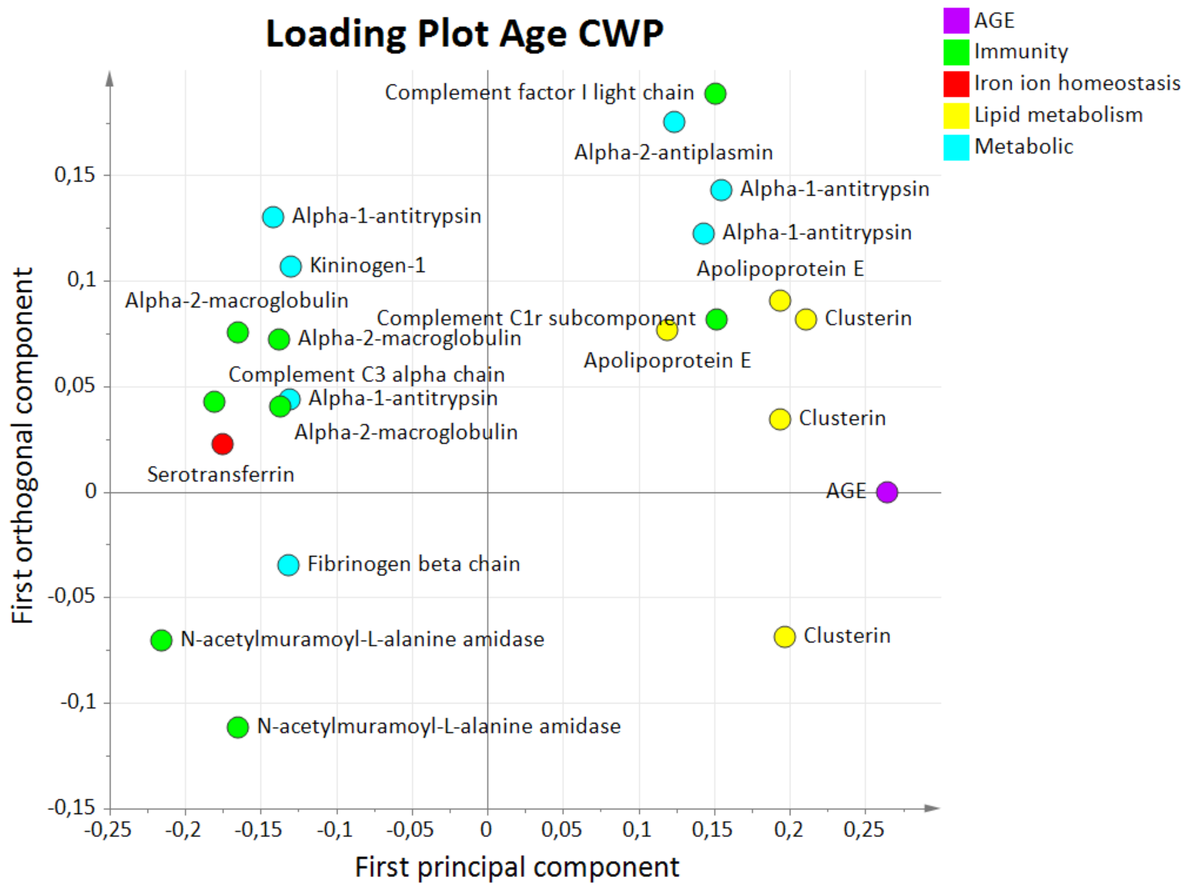

Supplement: Supplementary file 1 [file Image_1.pdf]
