# Supplementary material for: Plasma Protein Pattern Correlates With Pain Intensity and Psychological Distress in Women With Chronic Widespread Pain
Source: Front Psychol. 2018 Nov 29;9:2400. doi: 10.3389/fpsyg.2018.02400 (PMC6281753; doi:10.3389/fpsyg.2018.02400)
Supplement: Supplementary file 2 [file Table_1.pdf]

Supplementary Table S1. Mass spectrometry data of identified proteins. Spot number correspond to marked spots on the two dimensional gel in supplementary figure S1 and numbers in tables for each multivariate statistical model. MALDI-TOF and LC-MS analysis have been used to identify proteins, see method section for more details. Parts of the data have been published in previous studies, see references in column 10.
MW: molecular weight, p*I*: isoelectric point, MS: mass spectrometry.

Spot number	Protein name	Accession number	Theoretical MW/p*I*	Intensity	Score	MOWSE Score	Number of peptides	Sequence coverage (%)	Published MS data
2903	Alpha-1-antitrypsin	P01009	46 736/5.37	-	-	69.7E+3	9	25.1	
2904				-	-	2.44E+6	14	44.3	
2902				-	-	-	-	-	
3809				-	-	-	-	-	
3810				2.61E+09	323.31	-	24	61.5	
3420				9.91E+09	323.31	-	25	60.8	
2202				-	-	-	-	-	
2601	Alpha-1B-glycoprotein	P04217	54 253/5.36	3.01E+09	323.31	-	15	55.4	
2701				-	-	-	-	-	
3709				-	-	-	-	-	
2604	Alpha-2-antiplasmin	P08697	54 566/5.87	-	-	-	-	-	
3606				-	-	-	-	-	
3616				-	-	239.5E+3	11	30.8	
3617				-	-	-	-	-	
5821	Alpha-2-macroglobulin	P01023	163 290/6.03	6.23E+08	323.31	-	31	26.1	
5816				-	-	-	-	-	
5902				-	-	-	-	-	
5815				4.44E+07	323.31		16	13.6	
3406	Antithrombin-III	P01008	52 602/6.32	6.46E+08	323.31	-	20	44.6	
4522				9.58E+06	323.31	-	10	22.8	
3119	Apolipoprotein A-I	P02647	30 777/5.56	3.57E+09	323.31	-	20	72.7	
1051	Apolipoprotein C-II	P02655	11 284/4.64	1.00E+09	180.49	-	4	49.5	
1052	Apolipoprotein C-III	P02656	10 852/5.23	1.97E+08	323.31	-	3	20.8	

3103<br>4224<br>4225	Apolipoprotein E	P02649	36 154/5.65	3.67E+07<br>-<br>7.84E+08	323.31<br>-<br>323.31	-<br>-<br>-	15<br>-<br>14	46.7<br>-<br>42.3	
6407	Beta-2-glycoprotein 1	P02749	38 298/8.34	1.16E+08	323.31	-	9	30.4	
5723<br>6713	C4b-binding protein alpha chain	P04003	67 034/7.2	-<br>-	-<br>-	6.2E+3<br>3.68E+7	7<br>16	11.4<br>28.6	
3817<br>4833	Ceruloplasmin	P00450	122 206/5.4	-<br>-	-<br>-	92.1E+3<br>1.05E+6	14<br>10	15.6<br>12.5	[1]<br>[1]
8306	Chitinase-3-like protein 1	P36222	42 625/8.69	-	-	196.0	11	28	
114<br>131<br>132<br>1104<br>1113<br>3214	Clusterin	P10909	52 494/5.88	3.82E+08<br>7.85E+08<br>8.69E+08<br>1.34E+09<br>7.50E+08<br>4.06E+08	323.31<br>323.31<br>323.31<br>323.31<br>323.31<br>323.31	-<br>-<br>-<br>-<br>-<br>-	9<br>14<br>14<br>14<br>16<br>10	27.6<br>31.2<br>31.2<br>30.5<br>33.6<br>18.7	
4810<br>4809<br>5818<br>5819<br>5824<br>5825	Complement C1r subcomponent	P00736	80 119/5.8	-<br>-<br>-<br>-<br>-<br>-	-<br>-<br>-<br>-<br>-<br>-	3.0E+9<br>-<br>4.38<br>822.0E+3<br>1.71E+12<br>1.13E+11	10<br>-<br>4<br>12<br>19<br>22	20.6<br>-<br>8.4<br>15.4<br>30.6<br>33.0	[1]<br><br>[1]<br>[1]<br>[1]<br>[1]
1806	Complement C1s subcomponent	P09871	76 685/4.9	-	-	169.2E+3	14	21.9	[1]
6840<br>6842	Complement C3 alpha chain	P01024	103 956/5.18	1.63E+07<br>1.99E+07	323.31<br>323.31	-<br>-	15<br>20	13<br>16.7	
145	Complement C3c alpha chain fragment 2	P01024	39 488/4.79	-	-	6.34E+7	15	56.0	[1]
7216<br>8101<br>9101	Complement C4-B	P0C0L5	33073/6.37	-<br>-<br>-	-<br>-<br>-	5.2E+3<br>1.12E+7<br>3.19E+8	5<br>8<br>12	14.1<br>32.6<br>50.9	
9522	Complement C4-B beta chain	P0C0L5	71 678/8.69	3.01E+09	323.31	-	29	20.1	
6845	Complement component C7	P10643	93 519/6.1	-	-	105.8E+3	10	13.0	

7901	Complement factor B	P00751	85 534/6.7	-	-	1.19e+18	31	51.3	
1111	Complement factor I light chain	P05156	27 592/6.24	-	-	1.6E+3	6	32.0	[1]
8618	Fibrinogen alpha chain	P02671	94 972/5.70	-	-	-	-	-	
8619				2.87E+09	323.3E+0	323.3E+0	24	31.5	
8620				-	-	-	-	-	
8621				-	-	-	-	-	
8630				-	-	-	-	-	
8634				-	-	-	-	-	
8718				1.79E+09	323.31	-	23	28.9	
8719				4.39E+09	323.31	-	27	37.3	
9601				-	-	-	-	-	
9602				2.45E+09	323.31	-	25	32.8	
9208	Fibrinogen alpha chain fragment	P02671	91 358/5.79	4.07E+07	306.9	-	12	14.3	
7519	Fibrinogen beta chain	P02675	55 928/8.54	7.02E+09	323.31	-	25	68.4	
7524				4.73E+09	323.31	-	27	70.7	
8520				7.37E+07	323.31	-	11	33.2	
4301	Fibrinogen gamma chain	P02679	51 512/5.4	-	-	-	-	-	
4302				-	-	3.84e+13	19	53.4	[1]
4304				-	-	1.14e+16	18	59.2	[1]
6324	Ficolin-3	O75636	32 903/6.2	-	-	31.2E+3	6	20.1	[1]
1204	Haptoglobin	P00738	45 205/6.13	-	-	-	-	-	
2203				1.10E+09	323.31	-	12	37.9	
3105				6.62E+08	323.31	-	14	40.6	
4104				6.71E+08	323.31	-	14	40.9	
4316				-	-	-	-	-	
5319				-	-	-	-	-	
3209	Haptoglobin beta chain	P00738	27 265/6.32	-	-	35.6E+3	8	34.7	[1]
5833	Hemopexin	P02790	51 676/6.55	-	-	85	4	7	
4604				-	-	2.43e+9	16	44.8	
6608				-	-	104	9	19	

4503	Ig alpha-2 chain C region	P01877	36 527/5.7	-	-	481	5	18.8	[1]
6505				-	-				
6114	Ig kappa chain C region	P01834	11 765/5.6	-	-	1.1E+3	2	30.2	[1]
7120				-	-	-	-	-	
8135				-	-	-	-	-	
9006				4.18E+08	32.60	-	2	80.4	
510	Kininogen-1	P01042	71 957/6.34	4.95E+08	323.31	-	13	22.8	
2406				4.88E+08	323.31	-	21	29.8	
1414	Leucine-rich alpha-2-glycoprotein	P02750	38 177/6.45	4.99E+08	323.31	-	9	30	
1416				2.39E+08	323.31	-	7	24.8	
5721	N-acetylmuramoyl-L-alanine amidase	Q96PD5	62 217/7.25	-	-	-	-	-	
6602				-	31	-	1	1	[2]
7819	Plasminogen	P00747	90 570/7.0	-	-	-	-	-	
7820				-	-	-	-	-	
8822				-	-	6.00E+16	30	50.4	[1]
8823				-	-	5.94E+12	22	30.6	[1]
8901				-	-	-	-	-	
8909				-	-	-	-	-	
8910				-	-	9.09E+17	29	46.5	[1]
9901				-	-	-	-	-	
3717	Secretory immunoglobulin chain alpha	P99003	93 722/5.5	-	-	-	-	-	[3], [4], [5]
4713				-	-	-	-	-	
4807				-	-	-	-	-	
4808				-	-	-	-	-	
6731	Serotransferrin	P02787	77 063/6.81	1.79E+08	323.31	-	28	42.6	
6839				-	-	-	-	-	
7315				3.57E+08	323.31	-	27	42.7	
7743				9.89E+09	323.31	-	43	64.5	
4119	Serum amyloid P-component	P02743	25 387/6.10	4.69E+08	136.86	-	7	28.3	

2116	Transthyretin	P02766	15 887/ 5.5	-	-	97.5	4	30.0	[1]
9209	Unidentified	Unknown	-	-	-	-	-	-	
3408<br>3405	Vitamin D-binding protein	P02774	52 963/5.32	1.91E+09<br>-	323.31<br>-	-<br>-	15<br>-	48.1<br>-	

References

1. Wåhlén, K., Olausson, P., Carlsson, A., Ghafouri, N., Gerdle, B., & Ghafouri, B. (2017). Systemic alterations in plasma proteins from women with chronic widespread pain compared to healthy controls: a proteomic study. J Pain Res, 10, 797-809. doi:10.2147/jpr.s128597.
2. Ghafouri, B., Carlsson, A., Holmberg, S., Thelin, A., & Tagesson, C. (2016). Biomarkers of systemic inflammation in farmers with musculoskeletal disorders; a plasma proteomic study. *BMC Musculoskelet Disord, 17*(1), 206. doi:10.1186/s12891-016-1059-y
3. Artimo, P., Jonnalagedda, M., Arnold, K., Baratin, D., Csardi, G., de Castro, E., . . . Stockinger, H. (2012). ExPASy: SIB bioinformatics resource portal. *Nucleic Acids Research, 40*(W1), W597-W603. doi:10.1093/nar/gks400
4. Swiss Institute of Bioinformatics, SWISS 2D-PAGE, ExPASy Molecular Biology Server at https://world-2dpage.expasy.org/swiss-2dpage/
5. Anderson, L., & Anderson, N. G. (1977). High resolution two-dimensional electrophoresis of human plasma proteins. *Proc Natl Acad Sci U S A, 74*(12), 5421-5425.
