# Supplementary material for: Plasma Protein Pattern Correlates With Pain Intensity and Psychological Distress in Women With Chronic Widespread Pain
Source: Front Psychol. 2018 Nov 29;9:2400. doi: 10.3389/fpsyg.2018.02400 (PMC6281753; doi:10.3389/fpsyg.2018.02400)
Supplement: Supplementary file 3 [file Table_2.pdf]

Supplementary tables

Supplementary Table S2. **OPLS of HADS in CON group**. 12 proteins had a VIP >1. The proteins with highest VIP value (bold) were considered as important regressors for the model. SD: standard deviation; CON: control; CWP: chronic widespread pain; OD: optical density; HADS: hospital anxiety and depression scale; MW: molecular weight; p*I*: isoelectric point; VIP: variable influence on projection; OPLS: orthogonal partial least squares regression analysis.

Spot number	Protein Name	Accession number	Biological process	Experimental MW (kDa)/p*I*	VIP	p(corr)	OD CON (Mean ± SD)	OD CWP (Mean ± SD)	OD quotient mean	Alteration CWP vs CON
**8909**	**Plasminogen**	**P00747**	**Metabolic**	**151 / 6.65**	**1.43**	**0.45**	**322 ± 203**	**618 ± 375**	**1.92**	↑
**7820**	**Plasminogen**	**P00747**	**Metabolic**	**154 / 6.55**	**1.38**	**0.51**	**226 ± 154**	**269 ± 160**	**1.19**	↑
**9209**	**Unidentified**	**Unknown**	**Unknown**	**43 / 8.30**	**1.28**	**0.55**	**265 ± 177**	**303 ± 325**	**1.14**	↑
**3717**	**Secretory immunoglobulin chain alpha**	**P99003**	**Immunity**	**125 / 5.38**	**1.27**	**0.52**	**161 ± 131**	**143 ± 111**	**0.89**	↓
**3617**	**Alpha-2-antiplasmin**	**P08697**	**Metabolic**	**107 / 5.30**	**1.26**	**0.56**	**82 ± 108**	**197 ± 264**	**2.40**	↑
3405	Vitamin D-binding protein	P02774	Inflammatory	81 / 5.47	1.23	-0.49	228 ± 262	804 ± 753	3.53	↑
7216	Complement C4-B	P0C0L5	Immunity	46 / 6.13	1.16	0.42	865 ± 723	1001 ± 920	1.16	↑
8634	Fibrinogen alpha chain	P02671	Metabolic	99 / 7.11	1.12	0.50	447 ± 177	565 ± 263	1.26	↑
9101	Complement C4-B	P0C0L5	Immunity	44 / 7.02	1.11	0.44	4005 ± 2187	5426 ± 1584	1.35	↑
8822	Plasminogen	P00747	Metabolic	143 / 6.91	1.07	0.44	430 ± 292	745 ± 337	1.73	↑
8101	Complement C4-B	P0C0L5	Immunity	45 / 6.35	1.05	0.38	2511 ± 1437	3344 ± 1364	1.33	↑
7819	Plasminogen	P00747	Metabolic	143 / 6.73	1.04	0.28	325 ± 224	524 ± 280	1.61	↑

Supplementary Table S3. **OPLS model CWP and BMI.** 21 proteins had a VIP>1. The majority of the proteins belonged to metabolic process. The proteins with highest VIP value belonged fibrinogen and apolipoprotein A-I (marked in bold.) SD: standard deviation; CON: control; CWP: chronic widespread pain; OD: optical density; BMI: body mass index; MW: molecular weight; p*I*: isoelectric point; VIP: variable influence on projection; OPLS: orthogonal partial least squares regression analysis.

Spot number	Protein name	Accession number	Biological process	Experimental MW (kDa)/p*I*	VIP	p(corr)	OD CON (Mean ± SD)	OD CWP (Mean ± SD)	OD quotient mean	Alteration CWP vs CON
**9601**	**Fibrinogen alpha chain**	**P02671**	**Metabolic**	**107 / 6.91**	**1.52**	**0.82**	**2673 ± 995**	**3027 ± 1469**	**1.13**	↑
**3119**	**Apolipoprotein A-I**	**P02647**	**Lipid metabolism**	**26 / 5.51**	**1.50**	**-0.72**	**5972 ± 3744**	**3524 ± 1496**	**0.59**	↓
**8619**	**Fibrinogen alpha chain**	**P02671**	**Metabolic**	**103 / 7.23**	**1.39**	**0.73**	**2051 ± 758**	**2404 ± 1069**	**1.17**	↑
**4304**	**Fibrinogen gamma chain**	**P02679**	**Metabolic**	**71 / 5.66**	**1.36**	**0.72**	**16721 ± 6137**	**25746 ± 8738**	**1.54**	↑
**8620**	**Fibrinogen alpha chain**	**P02671**	**Metabolic**	**101 / 7.47**	**1.32**	**0.66**	**1558 ± 573**	**1727 ± 780**	**1.11**	↑
7519	Fibrinogen beta chain	P02675	Metabolic	87 / 6.48	1.31	0.61	15648 ± 4171	16419 ± 6543	1.05	↑
7524	Fibrinogen beta chain	P02675	Metabolic	89 / 6.25	1.30	0.61	6114 ± 1636	5914 ± 1533	0.97	↓
4522	Antithrombin-III	P01008	Metabolic	76 / 5.61	1.28	-0.62	1418 ± 4382	316 ± 347	0.22	↓
8719	Fibrinogen alpha chain	P02671	Metabolic	107 / 7.11	1.27	0.66	3726 ± 1058	4040 ± 1837	1.08	↑
4713	Secretory immunoglobulin chain alpha	P99003	Immunity	125 / 5.47	1.27	0.66	135 ± 124	129 ± 119	0.96	↓
5721	N-acetylmuramoyl-L-alanine amidase	Q96PD5	Immunity	109 / 5.80	1.26	-0.69	1007 ± 567	1073 ± 520	1.07	↑
9602	Fibrinogen alpha chain	P02671	Metabolic	105 / 7.23	1.18	0.62	1823 ± 582	2018 ± 1033	1.11	↑
114	Clusterin	P10909	Lipid metabolism	46 / 4.99	1.16	-0.65	1879 ± 1725	1621 ± 921	0.86	↓
8630	Fibrinogen alpha chain	P02671	Metabolic	105 / 6.91	1.16	0.61	1520 ± 563	1711 ± 884	1.13	↑
8718	Fibrinogen alpha chain	P02671	Metabolic	107 / 7.02	1.15	0.60	3548 ± 1494	3911 ± 2244	1.10	↑
4807	Secretory immunoglobulin chain alpha	P99003	Immunity	125 / 5.51	1.14	0.66	114 ± 101	149 ± 117	1.31	↑
9006	Ig kappa chain C region	P01834	Immunity	26 / 7.83	1.07	-0.65	14576 ± 6884	11668 ± 7034	0.80	↓
3103	Apolipoprotein E	P02649	Lipid metabolism	44 / 5.38	1.07	-0.65	1524 ± 455	1459 ± 353	0.96	↓
8621	Fibrinogen alpha chain	P02671	Metabolic	99 / 7.59	1.06	0.57	825 ± 277	1113 ± 695	1.35	↑
4302	Fibrinogen gamma chain	P02679	Metabolic	71 / 5.58	1.05	0.62	11371 ± 3908	17521 ± 5893	1.54	↑
8135	Ig kappa chain C region	P01834	Immunity	30 / 7.47	1.01	-0.49	18253 ± 5862	17229 ± 3745	0.94	↓

Supplementary Table S4. OPLS model of CWP and Age. In total 21 proteins had a VIP>1 and belonged to different biological processes. The proteins with highest VIP values are marked in bold and are considered as the most important regressors for the model. SD: standard deviation; CON: control; CWP: chronic widespread pain; OD: optical density; MW: molecular weight; p*I*: isoelectric point; VIP: variable influence on projection; OPLS: orthogonal partial least squares regression analysis.

Spot number	Protein Name	Accession number	Biological process	Experimental MW (kDa)/p*I*	VIP	p(corr)	OD CON (Mean ± SD)	OD CWP (Mean ± SD)	OD quotient mean	Alteration CWP vs CON
**132**	**Clusterin**	**P10909**	**Lipid metabolism**	**46 / 5.09**	**1.74**	**0.82**	**1569 ± 1625**	**1331 ± 986**	**0.85**	↓
**6602**	**N-acetylmuramoyl-L-alanine amidase**	**Q96PD5**	**Immunity**	**109 / 5.86**	**1.63**	**-0.84**	**1947 ± 1216**	**2065 ± 937**	**1.06**	↑
**1113**	**Clusterin**	**P10909**	**Lipid metabolism**	**45 / 5.17**	**1.60**	**0.76**	**1501 ± 1437**	**1545 ± 1275**	**1.03**	↑
**7315**	**Serotransferrin**	**P02787**	**Iron ion homeostasis**	**56 / 6.40**	**1.53**	**-0.68**	**397 ± 673**	**271 ± 565**	**0.68**	↓
**4225**	**Apolipoprotein E**	**P02649**	**Lipid metabolism**	**40 / 5.69**	**1.52**	**0.76**	**173 ± 208**	**348 ± 436**	**2.01**	↑
131	Clusterin	P10909	Lipid metabolism	47 / 4.99	1.43	0.77	1050 ± 539	1147 ± 806	1.09	↑
2904	Alpha-1-antitrypsin	P01009	Metabolic	151 / 5.36	1.38	0.60	64 ± 97	99 ± 67	1.55	↑
6842	Complement C3 alpha chain	P01024	Immunity	157 / 5.96	1.32	-0.70	485 ± 254	470 ± 240	0.97	↓
2903	Alpha-1-antitrypsin	P01009	Metabolic	151 / 5.32	1.30	0.56	57 ± 111	70 ± 47	1.23	↑
4809	Complement C1r subcomponent	P00736	Immunity	132 / 5.58	1.28	0.59	158 ± 101	238 ± 155	1.51	↑
8520	Fibrinogen beta chain	P02675	Metabolic	82 / 6.91	1.27	-0.51	272 ± 102	299 ± 159	1.10	↑
5721	N-acetylmuramoyl-L-alanine amidase	Q96PD5	Immunity	109 / 5.80	1.27	-0.64	1007 ± 567	1073 ± 520	1.07	↑
2902	Alpha-1-antitrypsin	P01009	Metabolic	145 / 5.32	1.21	-0.55	160 ± 178	129 ± 145	0.81	↓
1111	Complement factor I light chain	P05156	Immunity	48 / 5.22	1.19	0.59	293 ± 273	786 ± 333	2.68	↑
2406	Kininogen-1	P01042	Metabolic	81 / 4.99	1.14	-0.51	185 ± 108	248 ± 122	1.34	↑
3810	Alpha-1-antitrypsin	P01009	Metabolic	145 / 5.26	1.12	-0.51	251 ± 286	232 ± 201	0.92	↓
5902	Alpha-2-macroglobulin	P01023	Immunity	157 / 5.71	1.12	-0.64	139 ± 147	190 ± 219	1.37	↑
5815	Alpha-2-macroglobulin	P01023	Immunity	157 / 5.69	1.09	-0.54	161 ± 236	170 ± 183	1.06	↑
3616	Alpha-2-antiplasmin	P08697	Metabolic	105 / 5.32	1.07	0.48	94 ± 100	214 ± 160	2.28	↑
4224	Apolipoprotein E	P02649	Lipid metabolism	42 / 5.69	1.03	0.46	487 ± 503	525 ± 348	1.08	↑
5816	Alpha-2-macroglobulin	P01023	Immunity	157 / 5.78	1.02	-0.53	148 ± 231	157 ± 157	1.06	↑
